# Human–AI collectives most accurately diagnose clinical vignettes

**DOI:** 10.1073/pnas.2426153122

**Published:** 2025-06-13

**Authors:** Nikolas Zöller, Julian Berger, Irving Lin, Nathan Fu, Jayanth Komarneni, Gioele Barabucci, Kyle Laskowski, Victor Shia, Benjamin Harack, Eugene A. Chu, Vito Trianni, Ralf H. J. M. Kurvers, Stefan M. Herzog

**Affiliations:** ^a^Center for Adaptive Rationality, Max Planck Institute for Human Development, Berlin 14195, Germany; ^b^The Human Diagnosis Project, San Francisco, CA 94110; ^c^Department of Digital Humanities, University of Cologne, Cologne 50931, Germany; ^d^Harvey Mudd College, Claremont, CA 91711; ^e^Department of Politics and International Relations, Oxford University, Oxford OX13UQ, United Kingdom; ^f^Kaiser Permanente, Downey, CA 90242; ^g^Laboratory of Autonomous Robotics and Artificial Life & Collective Intelligence in Natural and Artificial Systems Lab, Institute of Cognitive Sciences and Technologies, Italian National Research Council, Rome 00159, Italy; ^h^Science of Intelligence Excellence Cluster, Technical University Berlin, Berlin 10587, Germany

**Keywords:** medical diagnostics, collective intelligence, large language models, health informatics, AI

## Abstract

AI systems, particularly large language models (LLMs), are increasingly being employed in high-stakes decisions that impact both individuals and society at large, often without adequate safeguards to ensure safety, quality, and equity. Yet LLMs hallucinate, lack common sense, and are biased—shortcomings that may reflect LLMs’ inherent limitations and thus may not be remedied by more sophisticated architectures, more data, or more human feedback. Relying solely on LLMs for complex, high-stakes decisions is therefore problematic. Here, we present a hybrid collective intelligence system that mitigates these risks by leveraging the complementary strengths of human experience and the vast information processed by LLMs. We apply our method to open-ended medical diagnostics, combining 40,762 differential diagnoses made by physicians with the diagnoses of five state-of-the art LLMs across 2,133 text-based medical case vignettes. We show that hybrid collectives of physicians and LLMs outperform both single physicians and physician collectives, as well as single LLMs and LLM ensembles. This result holds across a range of medical specialties and professional experience and can be attributed to humans’ and LLMs’ complementary contributions that lead to different kinds of errors. Our approach highlights the potential for collective human and machine intelligence to improve accuracy in complex, open-ended domains like medical diagnostics.

## Significance

Large language models (LLMs) have great potential for high-stakes applications such as medical diagnostics but face challenges including hallucinations, biases, and lack of common sense. We address these limitations through a hybrid human–AI system that combines physicians’ expertise with LLMs to generate accurate differential medical diagnoses. Analyzing over 2,000 text-based medical case vignettes, hybrid collectives outperform individual physicians, standalone LLMs, and groups composed solely of physicians or LLMs, by leveraging complementary strengths while mitigating their distinct weaknesses. Our findings underscore the transformative potential of human–AI collaboration to enhance decision-making in complex, open-ended domains, paving the way for safer, more equitable applications of AI in medicine and beyond.

Diagnostic errors are among the most pressing issues in medical practice (1–3), causing an estimated 795,000 deaths and permanent disabilities in the United States alone each year (4). Reducing diagnostic errors—without incurring substantially higher costs—is essential to improve patient outcomes worldwide. This challenge has motivated a recent surge in diagnostic technologies within the field of health informatics, which exploit AI to interpret medical records, tests, and images (5, 6). Deep learning approaches in medical imaging have shown great promise. Notable examples include mammography interpretation, cardiac function assessment, and lung cancer screening, some of which have progressed beyond the testing phase and entered clinical practice (7–9).

Recent years have also witnessed the rise of AI foundation models, especially large language models (LLMs), which show remarkable abilities to process natural language, providing accurate answers to questions in almost any domain, including medicine (10–12). However, a recent meta-analysis (13) found that physicians often outperform LLMs, and that LLMs differ vastly in performance, also between medical specialties. While LLMs’ performance in the medical domain keeps improving (12), their deployment in clinical practice remains challenging due to the risk of errors [caused by, e.g., hallucinations (14–17), biases (18, 19), and lack of common sense (20)] and concerns about their trustworthiness (21). As these shortcomings may reflect inherent limitations of LLMs (22), developing more sophisticated architectures or using more data or more human feedback may not sufficiently address these shortcomings. The tension between the vast potential of AI-based solutions and the challenges of real-world deployment is not limited to medical diagnostics. It is also apparent in other domains, especially those involving high-stakes decisions whose effects are not immediate, such as strategies to address climate change (23).

Here, we present an approach that complements AI responses with human expert knowledge in open-ended medical diagnostics. This method, which combines AI with a collective intelligence (CI) approach, benefits from the diversity of solutions provided by humans and LLMs. CI approaches harness the contributions of multiple experts to reduce errors and find creative solutions to complex problems (24, 25). In medical diagnostics, several studies have found that the collective solution of multiple diagnosticians outperforms the average individual across a range of medical contexts (26–32). These studies have focused on binary or small-scale decision problems (e.g., detecting a specific condition), but CI has also proved successful in open-ended medical problems. While in earlier studies the contributions of individual experts are manually harmonized and aggregated into collective diagnoses (33), more recently this approach has been fully automatized. Specifically, medical knowledge graphs and natural language processing methods are leveraged to harmonize the free-text contributions of individual experts (34), which can differ significantly due to the open-endedness of the solution space (35).

In a similar vein, it has been postulated that AI can enhance human collective intelligence (36, 37). Hybrid systems that integrate state-of-the-art LLMs as peers in a mixed human–AI collective hold promise for addressing complex decision problems such as medical diagnostics. AI can provide complementary information without perpetuating the errors and biases of human peers. At the same time, the diagnostic process is not entirely outsourced to artificial systems, making it possible to benefit from human experts’ ability to think outside the box, recognize context, and handle contentious evidence, thus mitigating the risks of LLMs.

Combining the contributions of multiple humans and multiple LLMs is, however, not straightforward. Although many studies have explored how to combine multiple AI models [e.g., ensemble learning is an established practice in machine learning (38, 39)], little is known about how to best combine the outputs of multiple LLMs (but see refs. 40–43 for specific use cases), or how to combine the responses of multiple LLMs with those of human experts, particularly in open-ended domains. In this study, we develop a general-purpose method to combine the responses produced by both human experts and LLMs. Applying this method to a set of over 40,000 diagnoses, we show that hybrid human–AI collectives outperform human-only and LLM-only collectives in diagnosing text-based clinical vignettes across a variety of medical specialties and levels of professional experience. Additionally, we demonstrate that when LLMs fail, physicians often provide correct diagnoses, thus highlighting the crucial importance of maintaining expert involvement, even in the presence of an ensemble of powerful AIs.

## 1. Medical Cases, Human Data and LLM Responses

**Fig. 1.**
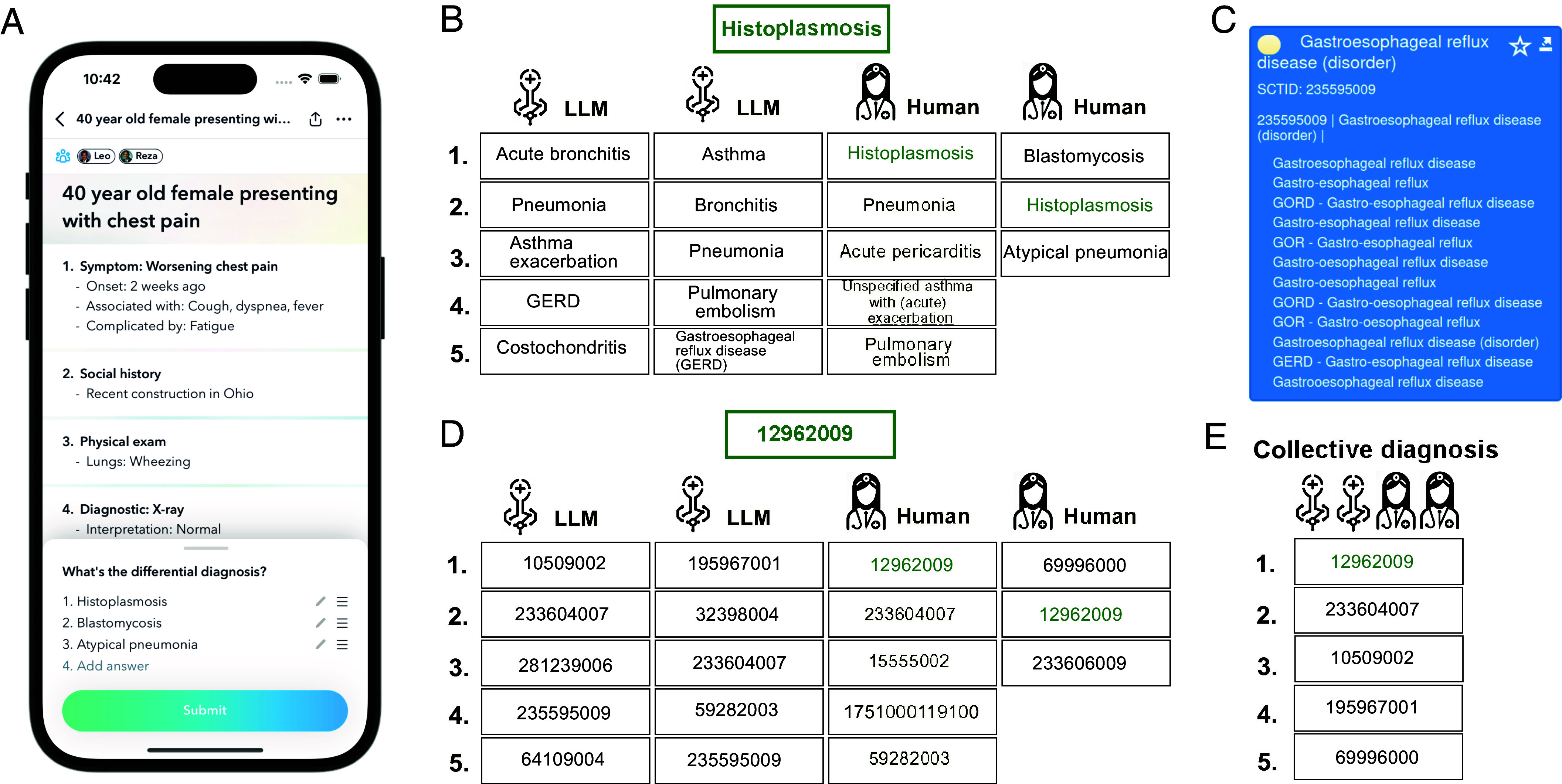
Illustration of the hybrid collective intelligence process, which combines human diagnoses with LLM outputs to arrive at a collective differential diagnosis. (*A*) Screenshot of the interface that human users see when diagnosing a patient case on the Human Dx platform via a mobile device. The information provided can include a patient’s symptoms, test results, and medical record. Users can uncover this information piece by piece and update their diagnosis accordingly. In this analysis, we only consider users’ final differential diagnosis. The same information shown to human users is also given to LLMs as part of a prompt (*Materials and Methods*). (*B*) An illustrative example of the open-ended text responses given by users and LLMs. Next, extending a method presented in ref. 34 (*Materials and Methods* and *SI Appendix*, Fig. S1), each single diagnosis is subjected to several processing steps for standardization, after which it is assigned a unique ID in the SNOMED CT healthcare terminology. (*C*) Example of a SNOMED CT entry. Crucially, all listed synonyms are matched to the same SNOMED CT ID. (*D*) Diagnoses of humans and LLMs after the matching step. (*E*) Collective diagnosis after aggregating the diagnoses from humans and LLMs. In this aggregation, LLMs and humans are assigned different weights based on their performance in the training fold. The rank r of a diagnosis in a differential diagnosis is taken into account through a 1/r scoring rule (*Materials and Methods*).

The empirical basis for this work is a dataset from the Human Diagnosis Project (Human Dx), an online collaborative platform for medical professionals and trainees. Users from around the world can register on the platform, submit cases, review case details, and provide diagnoses. The cases submitted are published only if approved by an editorial board of licensed medical professionals. Each case is presented as a vignette mimicking information that physicians encounter in real-world practice and containing patient information such as symptoms, medical records, and clinical test results (Fig. 1). When responding to a case, users can provide either a single diagnosis or a ranked list, commonly known as a differential diagnosis, either as free text or by selecting from a medical taxonomy with an autocomplete feature that activates as they type (see Fig. 1*A* for an illustration of the user interface). We refer to this response as a differential diagnosis, whether it contains one or multiple diagnoses. Once the user has submitted their differential diagnosis, they are shown the gold-standard solution as provided by cases’ authors and vetted by an expert panel, which may consist of one or several diagnoses (*Materials and Methods*). For our main analyses, we used a set of 2,133 medical cases and 40,762 differential diagnoses from qualified physicians with different levels of professional experience (*Materials and Methods*). In *SI Appendix*, we additionally present results of the same analyses for medical students.

To compare and combine the human diagnoses with LLM outputs, we provided the same set of case vignettes to five commercially available or open-source state-of-the-art LLMs (Anthropic Claude 3 Opus, Google Gemini Pro 1.0, Meta Llama 2 70B, Mistral Large, and OpenAI GPT-4) and prompted the models to provide the five most probable diagnoses, ordered by their probability of being correct (*Materials and Methods*).

## 2. Harmonizing, Aggregating, and Evaluating Open-Ended Answers from Doctors and LLMs

The process of aggregating human judgments and LLM outputs into a collective diagnosis is illustrated in Fig. 1. In brief, each diagnosis is assigned a weighted score, which is determined by considering both its rank in individual diagnostic lists (with higher-ranked diagnoses receiving more weight) and the accuracy of the source providing the diagnosis. To estimate this accuracy, we used a repeated five-fold cross-validation approach in which onefold was used as a training fold to optimize LLM prompting and compute separate weights for LLMs and human experts. Since many individual human experts only diagnosed one or a few cases, we did not assign distinct weights to individual experts. Instead, all human experts were assigned a single weight based on their collective diagnostic performance on the training fold. In contrast, because LLMs provided diagnoses for all cases, we were able to learn separate weights for each LLM. The remaining four folds were then used to evaluate the performance of these weighted collective diagnoses (see *Materials and Methods* and *SI Appendix*, Fig. S1 for further details).

In order to make the open-ended diagnoses of users and LLMs comparable and uniquely identifiable, we extended the method described in ref. 34, which maps free-text diagnoses to concepts (and their unique IDs) in the Systematized Nomenclature of Medicine Clinical Terms (SNOMED CT) (44) (*Materials and Methods*). SNOMED CT is a comprehensive clinical terminology and coding system designed to standardize the representation of medical concepts and support the accurate communication of clinical information in healthcare. After matching diagnoses to SNOMED-CT concepts, the generation of the collective differential diagnoses proceeds exploiting the SNOMED-CT IDs (Fig. 1).

Depending on the use case for an aggregated collective diagnosis, some performance metrics might be more suitable than others. For example, if the differential diagnosis of an LLM (ensemble), a human collective, or a hybrid collective serves as a consideration set to support the decision of a human physician, it may be sufficient that the correct solution is included in the differential diagnosis at all, and less important that it is ranked first. Therefore, we report several accuracy metrics, including top-5, top-3, and top-1 accuracy, where a differential diagnosis is evaluated as correct if the correct diagnosis is among the top five, top three, or top one diagnoses, respectively (and the accuracy is the proportion of such cases). For the fraction of cases where a case author has stated several diagnoses as correct (34%), a nominated diagnosis is considered correct if it matches any of the correct diagnoses. Additionally, we report the mean reciprocal rank (MRR) (45), a well-established performance metric in the field of information retrieval, defined as

[1] $$\;\text{MRR}=\frac{1}{C}\sum_{i=1}^{C}\frac{1}{\;{\text{r}}_{i}},$$

where C corresponds to the number of cases on which the metric is evaluated and $r_{i}$ is the rank of the first occurrence of a correct answer in the final list for case i. Note that if $r_{i}>5$ or if the correct diagnosis is not present in the ranking, we set $r_{i}=\infty$ so that the contribution of case i to the MRR is null.

## 3. Aggregating LLMs Increases Performance in Open-Ended Medical Diagnostics

**Fig. 2.**
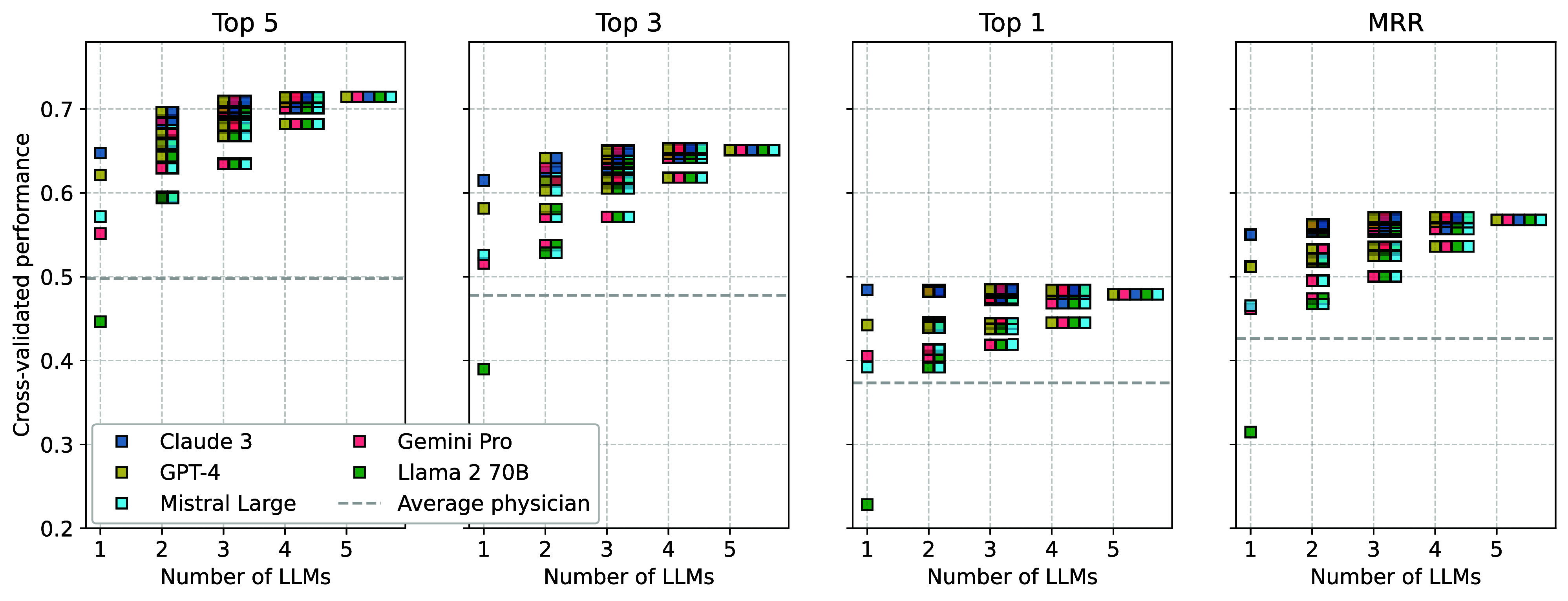
Cross-validated performance of five individual LLMs (Anthropic Claude 3 Opus, OpenAI GPT-4, Mistral Large, Google Gemini Pro 1.0 and Meta Llama 2 70B) and ensembles of all possible combinations of LLMs. Panels show performance for four outcome metrics (y axes): Top-k indicates the proportion of cases for which the correct diagnosis was among the k top-ranked diagnoses (for k={1,3,5}); MRR shows the mean reciprocal rank of correct diagnoses across cases (Eq. **1**). The x axis shows the number of LLMs in an ensemble. The horizontal dashed line shows the average individual performance of the physicians (i.e., first averaged within cases, then across all cases). Some of the ensembles overplot each other (see *SI Appendix*, Figs. S2 and S3 for the full Bayesian posterior distributions, including 95% credible intervals).

We start by presenting the cross-validated results for the baseline performance of the five individual LLMs and all possible LLM ensembles. As Fig. 2 shows, the individual LLMs differed notably in performance, but aggregating multiple LLMs into ensembles generally increased diagnostic accuracy. The ensembles performed much better than the worst individual LLM and generally as well as, or better than, the best individual LLM. For clarity, error bars are omitted here, but the full Bayesian posterior distributions, including 95% credible intervals, are provided in *SI Appendix*, Figs. S2 and S3). *SI Appendix*, Fig. S4 shows Bayesian performance comparisons between the all-LLM ensemble and individual LLMs. For top-5 accuracy, the ensemble of all LLMs combined clearly outperformed each LLM individually, and this result held across the five most common medical specialties in our data (cardiology, gastroenterology, pulmonology and respirology, neurology, and infectious diseases; see *SI Appendix*, Fig. S5). The same held for top-3 accuracy and MRR when comparing performance across all cases and for four of the five medical specialties (*SI Appendix*, Fig. S5). For top-1 accuracy, the ensemble of all LLMs performed better than four of the five individual LLMs and approximately at the level of the best-performing LLM (*SI Appendix*, Fig. S4). Whether or not it is advisable to aggregate several LLMs may therefore depend on the target metric, but if the purpose is to provide a consideration set to support the decision of a human physician (e.g., top-5 diagnoses), then LLM ensembles have the greatest potential.

To put the LLMs’ performance into perspective, *SI Appendix*, Fig. S6 shows the percentage of physicians who were outperformed by (and/or tied with) individual LLMs and LLM ensembles across the set of cases they had solved. This percentage was highest for an LLM ensemble incorporating all five LLMs (i.e., strictly outperformed 85% of physicians and outperformed or tied with 93% of physicians). Comparing the individual LLM performance with that of the human users showed that four of the five LLMs outperformed the average physician.

## 4. Human–AI Collective Intelligence Outperforms Both Humans and LLMs

**Fig. 3.**
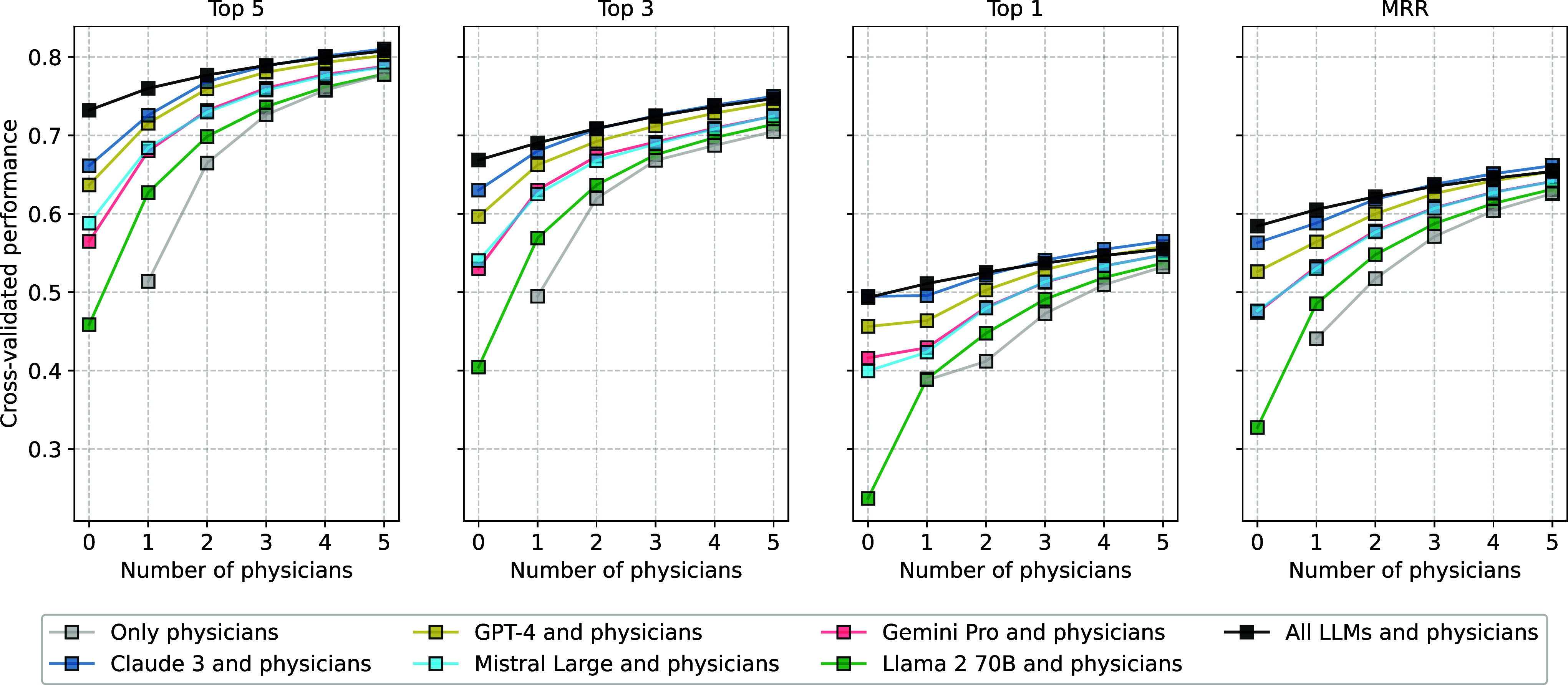
Cross-validated performance of human-only ensembles and hybrid ensembles of humans and LLMs. Panels show performance for four outcome metrics (y axes): Top-k indicates the proportion of cases for which the correct diagnosis was among the k top-ranked diagnoses (for k={1,3,5}); MRR shows the mean reciprocal rank of correct diagnoses across cases (Eq. **1**). The individual performance of the five LLMs (and their combined performance in an all-LLMs ensemble) is shown as the *Left*-most square of each color in each panel. The x axis shows the number of humans added to individual LLMs or to an all-LLMs ensemble. Some of the ensembles overplot each other (see *SI Appendix*, Figs. S7 and S8 for the full Bayesian posterior distributions, including 95% credible intervals).

Next, we test the complementarity of human and LLM solutions in a hybrid CI approach. Fig. 3 shows the cross-validated performance when combining the diagnoses of multiple physicians (human-only ensembles as a baseline) with any one of the five individual LLMs or with all LLMs. Full Bayesian posterior distributions are provided in *SI Appendix*, Figs. S7 and S8). For the baseline of human-only ensembles, increasing the number of physicians increased diagnostic accuracy, with greater marginal increases in accuracy for smaller than for larger group sizes. These results are in line with earlier findings from a smaller set of Human Dx cases (33, 34).

Crucially, adding one LLM to the human diagnoses consistently increased performance for both individual physicians and human-only ensembles of different sizes, with the largest increase attained when adding the best-performing individual LLM or an all-LLM ensemble. For top-5 and top-3 performance metrics, adding the all-LLM ensemble was as good as or better than adding the best-performing LLM. For top-1 accuracy and MRR, adding either the best-performing LLM or the all-LLM ensemble yielded the best results—which of the two depended on the size of the human group. Even adding the worst-performing LLM, which by itself performed worse than the average individual physician, generally led to a slight increase in performance across all metrics. *SI Appendix*, Figs. S9 and S10 show Bayesian performance comparisons for hybrid ensembles versus individual LLMs and *SI Appendix*, Figs. S11 and S12 for physician ensembles versus hybrid ensembles with the same number of humans.

From the perspective of human-only ensembles, comparing the performance of ensembles of n humans with that of hybrid ensembles of n−1 humans plus one LLM (i.e., the same overall group size of n inputs) showed that adding either the best or second-best LLM or the all-LLM ensemble to a human-only ensemble outperformed adding another human (for Bayesian performance comparisons see *SI Appendix*, Fig. S13; depending on the accuracy metric and group size, this finding also tended to hold for the third- and fourth-best LLM; Fig. 3). From the perspective of individual LLMs or an all-LLM ensemble, adding one or more human(s) increased performance; this increase was most pronounced for the worst-performing LLMs. As *SI Appendix*, Figs. S14 and S15 show, these results held across the five most common medical specialties in our data and for medical students.

## 5. Complementarity of Human- and LLM-Generated Diagnoses

The results presented in Fig. 3 suggest complementarity of physicians and LLMs in diagnosing open-ended medical problems. However, given that most LLMs outperform the average individual physician, how can adding a single physician to an individual LLM—or even to an ensemble of LLMs—increase diagnostic accuracy? The key answer to this question is that humans and LLMs make different kinds of errors. The literature on both CI (46, 47) and machine ensembles (38, 48) recognizes that the less correlated the errors of its members are, the more successful the ensemble will be.

**Fig. 4.**
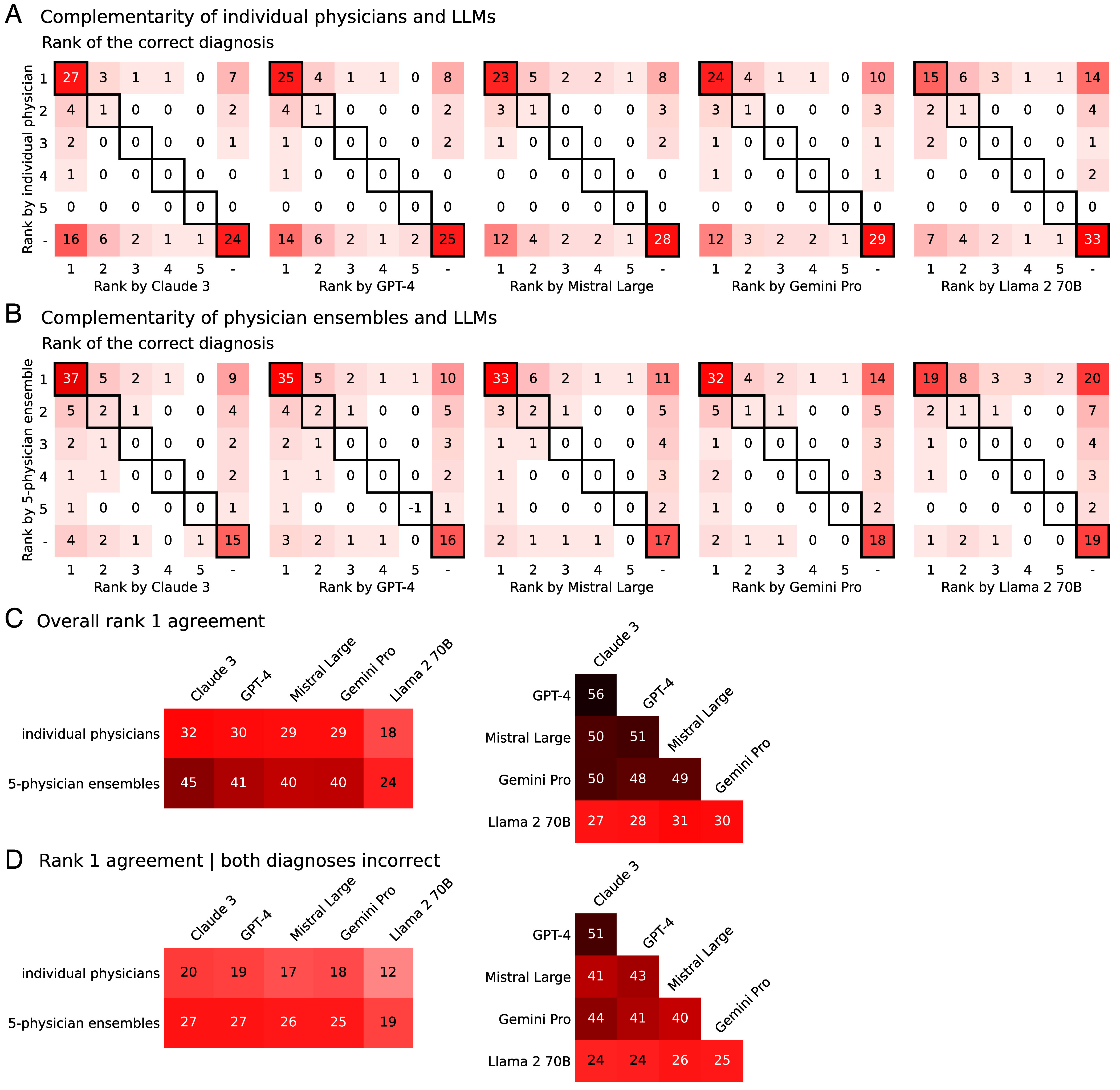
Complementarity of solutions from individual humans and human-only ensembles and LLMs. Panels (*A* and *B*) show, for each of the five LLMs, matrices with the percentages of cases for all 36 combinations of the LLM (x axis) and humans (y axis) assigning the correct diagnosis a particular rank (i.e., rank 1, 2, 3, 4, 5, or not ranked). (*A*) Results for individual physicians. (*B*) Results for five-physician human-only ensembles. The highlighted diagonal indicates cases where an LLM and the humans assigned the correct diagnosis the same rank. Panels (*C* and *D*) show the percentage of cases in which the same diagnoses were assigned rank one, comparing individual physicians and 5-physician ensembles to LLMs (*Left* side), and different LLMs to each other (*Right* side). (*C*) Overall rank one agreement, regardless of whether the correct diagnosis was included. (*D*) Rank one agreement when both diagnosticians were incorrect. Results were extracted from the ten-times repeated cross-validation procedure by recording the frequencies with which physicians and LLMs assigned the same or a different rank to either the correct or an incorrect diagnosis, averaged across all cases and the five folds (*Materials and Methods*). Note that due to rounding to integers, there may be small inconsistencies when summing rows or columns across matrices or when comparing sums of values to respective percentages reported in the main text.

Fig. 4*A* shows the percentage of cases in which individual physicians and LLMs placed the correct diagnosis on the same rank or both did not rank the correct diagnosis (highlighted diagonal cells) and the percentage of cases in which individual physicians and LLMs placed the correct diagnosis on different ranks (or it was only mentioned by either a physician or an LLM; all other cells). The results show that individual physicians and LLMs did not assign the correct diagnosis to the same rank in a substantial number of cases (range across LLMs: 46% to 51%). Crucially, when LLMs did not list the correct diagnosis at all (range across LLMs: 34% to 54%; *Right*-most columns), individual humans did mention it in a substantial number of cases (range across LLMs: 30% to 38%; *Right*-most columns excluding *Bottom*-*Right* cells), most frequently ranking it first (range across LLMs: 20% to 26%, *Top*-*Right* cells). In other words, diagnoses missed by LLMs were often made by individual physicians, frequently in first place. Thus, although individual physicians performed worse overall than most LLMs (Fig. 2 and *SI Appendix*, Fig. S6), in a substantial number of cases, they were able to compensate for the LLMs’ errors. Similarly, when individual humans did not list the correct diagnosis at all (49%, *Bottom* rows), LLMs did in a substantial number of cases (range across LLMs: 31% to 51%; *Bottom* columns excluding *Bottom*-*Right* cells), most frequently ranking it first (range across LLMs: 15% to 33%, *Bottom*-*Left* cells).

Fig. 4*B* shows the same analysis for five-physician collectives and LLMs. Given that human collectives outperformed individual humans (Fig. 3), the diagnoses given by five-physician collectives are even more complementary to the LLMs than are the ones given by individual physicians. When LLMs did not list the correct diagnosis at all (range across LLMs: 34% to 54%; *Right*-most columns), human ensembles did so in the majority of cases (range across LLMs: 55% to 65%; *Right*-most columns excluding *Bottom*-*Right* cells), most frequently ranking it first (range across LLMs: 27% to 36%, *Top*-*Right* cells). Intriguingly, the opposite pattern was less pronounced. When human-only ensembles did not list the correct diagnosis at all (22%; *Bottom* rows), LLMs did so in only the minority of cases (range across LLMs: 17% to 32%). For a similar complementarity analysis of LLMs with respect to each other, see *SI Appendix*, Fig. S16.

Fig. 4 *C* and *D* shows how often individual physicians, five-physician ensembles, and LLMs agree with each other on their top-ranked diagnosis. LLMs agree more among themselves than with physicians (Fig. 4*C*) and this difference is particularly pronounced in situations where both human and LLM diagnoses are incorrect (Fig. 4*D*). Furthermore, when humans and LLMs both make errors (Fig. 4*D*), they are less likely to assign the same incorrect diagnosis to the first rank compared to their respective overall agreement rate (which includes cases where either or both have ranked the correct diagnosis first; Fig. 4*C*). *SI Appendix*, Figs. S17 and S18 show that the above conclusions about error diversity also hold when considering the full range of ranks 1 to 5. This error diversity is crucial for a CI approach to be effective, and it is significantly more pronounced among hybrid pairs of a physician and an LLM compared to between pairs of different LLMs. Our key finding here is that in a collective aggregation scheme based on (weighted) majority voting, this error diversity ensures that correct diagnoses accumulate more frequently than incorrect ones, allowing the correct solutions to rise to the top of the collective differential diagnosis.

## 6. Discussion

Our results demonstrate the potential of combining human medical expertise with LLMs to enhance accuracy and reduce errors in open-ended medical diagnostics. Integrating the differential diagnosis of a single human diagnostician with the output of a single LLM yielded a better performance than either alone. Adding an LLM to multiple physicians’ diagnoses also improved performance in nearly all scenarios. The individual accuracy of the LLM influenced the performance gain, with the highest gain from the best-performing LLM. But even the worst-performing LLM, which was less accurate than the average human, showed positive effects.

Taking an LLM perspective, also the performance of LLMs could be boosted by adding human judgements. Adding a single physician increased performance for all LLMs even though individual physicians, on average, performed worse than most LLMs; and LLM performance increased steadily with adding more humans. The increase in performance was highest for the worst-performing LLM and lowest for the best-performing LLM.

An important component of (hybrid human–machine) CI is that different users or machines produce independent and diverse errors (46, 47, 49, 50). We find that humans and LLMs indeed make complementary errors that disperse throughout the vast solution space, while correct diagnoses accumulate and converge when integrating human and LLM diagnoses.

Previous work has shown the potential of AI and CI individually, and their hybrid combination for problems with well-defined, small solution spaces (e.g., categorization, probabilistic forecasting, numerical estimation) (51–54). Here, we showed that these results can be generalized to open-ended problems covering a vast solution space (there are more than 360,000 unique medical concepts of which more than 83,000 are tagged as disorders in the March 2023 international edition of SNOMED CT that we used), by using a general-purpose method to automatically harmonize and aggregate the solutions generated by humans and LLMs. While we demonstrated this method in the domain of medical diagnostics, we believe that our approach can be generalized to different applications for which structured domain knowledge is available, allowing the harmonization and principled aggregation of human expert judgements and LLM responses [e.g., climate change adaptation management (35)].

## 7. Limitations and Future Research

While our study demonstrates the potential of hybrid human–AI systems in medical diagnostics, further research is necessary to ensure the safety, reliability, efficacy, and ethical deployment of this technology in real-world clinical settings.

For instance, although vignette-based studies represent a validated and accepted paradigm for the study of diagnostic decision-making processes in medicine (55), it remains an open question as to how well our method translates to actual clinical practice. This concern has recently been echoed in critiques of current LLM evaluation practices, which argue that benchmark datasets derived from medical licensing exams fail to capture the complexity and ambiguity of real-world clinical tasks (56). Moreover, our case vignettes were selected by an expert panel at Human Dx, and users may flag suspicious cases for removal from the Human Dx platform. While all vignettes are designed to simulate real-world scenarios, the editorial team may prioritize cases based on their educational value and perceived interest to the user base. Consequently, this selection process may have excluded very difficult or rare cases, while also underrepresenting very common and straightforward cases typically encountered in clinical practice. This could help explain the relatively low top-1 diagnostic accuracy among physicians, compared to the error rates generally reported in outpatient care and emergency medicine (57–59). As a consequence, the improvement in accuracy observed across different ensembles likely represents an upper bound estimate of the potential benefit that could be expected in clinical practice. Future work could consider more ecologically valid or representative ways of selecting cases.

Furthermore, our analyses do not consider the consequences of the treatments implied by the diagnoses. Future work could study whether our proposed approach alters the likelihood of arriving at a potentially beneficial (or harmful) treatment. Such research must consider the decision context, as the recommended or accessible treatments may vary depending on the cultural, regional, and institutional circumstances, as well as the patients’ health insurance plan (e.g., ref. 60).

Finally, our study was not designed to address risks related to fairness and equity (see, e.g., refs. 61–66). For example, LLMs have been shown to perpetuate race-based medicine in their responses (18). This finding suggests that the clinical medical knowledge encoded in LLMs (12) is tainted by racism, which can leak into medical diagnoses, resulting in worse health outcomes for disadvantaged groups. Future work should directly study the extent to which the integration of humans and LLMs mitigates bias or amplifies biases shared among humans and LLMs in medical diagnostics (see also refs. 67 and 68).

More generally, taking a human-centered approach when designing hybrid systems is essential to compensate for the lack of transparency of AI models and for building trust among all affected stakeholders (69–72). Such an approach may help identify and mitigate some of the problems of LLMs or hybrid systems already during the design stage.

Future research could build on our approach in several ways. First, although we used a systematic prompt engineering approach, more sophisticated techniques have been developed that could further boost accuracy [e.g., tree of thought (73, 74), or self-consistency with temperature/top-p sampling (75)]. Applying sophisticated multilevel prompt-engineering techniques to generalist foundation models can improve performance and even outperform fine-tuned models for the medical domain (76). Second, vignettes could be classified into categories (e.g., medical specialties, number and type of case findings), and using tailored few-shot examples within these categories when prompting LLMs or adjusting weights for LLMs based on these categories may further boost accuracy. Additionally, more sophisticated weighting techniques could be tested that adjust weights based on fairness, or LLM biases (66). Third, we only considered text-based cases; future work could test the diagnostic performance of large multimodal models (and hybrid human–AI ensembles) on, for example, images (e.g., X-rays or histopathological images) or sounds (e.g., auscultation) alongside the textual information (10, 11). Forth, future work could further explore the potential of hybrid CI with nonexperts. *SI Appendix*, Fig. S15 demonstrates that hybrid ensembles of LLMs and medical students were able to outperform individual physicians and even groups of physicians. Boosting the performance of less qualified individuals by leveraging LLMs might have particular potential for underserved regions where access to experts is limited. Finally, while our study provides a proof-of-principle demonstrating the potential of hybrid collective intelligence for medical diagnostics, further research is needed to explore how our findings can be translated into clinical practice, for example in the form of a clinical decision support system. Future experiments could test whether using the aggregated responses from human-LLM ensembles as recommendations to the physician responsible for the final diagnosis influences the final diagnosis and increases accuracy. There are numerous studies demonstrating the effectiveness of clinical decision support (68, 77–80), and known factors influencing advice uptake are the timing of advice (81, 82) as well as automation bias (83, 84) and algorithmic aversion (85). However, more research is needed to understand the best setup for efficient hybrid collective intelligence decision support. One possibility for efficient use of human expert advice are fast and frugal approaches (86) through hybrid confirmation trees (87), where additional human expert advice is requested only when the initial human diagnosis and the diagnosis by an LLM ensemble disagree or when the diagnostician’s confidence is low.

## 8. Conclusion

Our study demonstrates the power of hybrid human–AI collectives in diagnosing text-based clinical vignettes, highlighting their potential relevance to general clinical practice. Hybrid collectives outperform both individual human experts and LLMs (as well as human-only and LLM-only collectives) in generating accurate differential diagnoses. This superior hybrid performance is a direct consequence of physicians and LLMs making different kinds of errors: When LLMs missed the correct diagnosis, individual physicians often contributed the correct diagnosis, rescuing the hybrid performance.

Recent years have seen a surge of research and publications on the potential of LLMs [e.g., in medical diagnostics; (12)]. However, in both science and public discourse, there is increasing concern about the lack of safeguards to ensure the safety, quality, and equity of LLM-based systems (21). LLMs, despite their impressive capabilities, hallucinate (14–17), lack common sense (20), and are biased (18, 19)—shortcomings that may reflect LLMs’ inherent limitations (22) and may thus not be remedied by more sophisticated architectures, more data, or more human feedback.

We posit that the time has come for a second wave of research on LLMs (and AI in general) that is no longer content to showcase what LLMs can do, propose technical approaches to fix their flaws (e.g., ref. 17), and speculate about how human oversight could be implemented. Rather, it is crucial to study how to leverage the complementary strengths of humans and AI by combining the experience and common sense of experts with the vast information processed by LLMs. In addition to technological solutions aimed at addressing problems inherent in an AI system [e.g., using retrieval-augmented generative AI to try addressing hallucinations; (17)], incorporating complementary human intelligence can help mitigate the risks of LLMs in ways that purely technological solutions may not ever be able to.

## 9. Materials and Methods

### 9.1. Human Dx: Medical Diagnostics Cases and Data from Human Solvers.

For our analyses, we used a dataset of 2,133 medical cases with a total of 40,762 diagnoses provided by medical experts through the user interface of the Human Dx app (Fig. 1*A*). Beforehand, we excluded from our analyses all diagnoses that were incomplete due to submission errors or connectivity issues. We also excluded the diagnoses of users who bypassed the onboarding process and of “shadow banned” users, who were permitted access to the platform but excluded from analyses due to unhelpful behavior (e.g., submitting diagnoses consisting of random characters or using profanities). Test accounts belonging to two Human Dx staff members were also excluded. Finally, we excluded cases containing images (as not all of the LLMs were able to process these). The medical experts consisted of 1,370 attending physicians (37.3%), 139 fellows (3.8%), and 2,160 resident physicians (58.9%), representing senior doctors, doctors undergoing specialized training, and doctors in training, respectively. Note that this tenure information is based on self-reports by the users. As *SI Appendix*, Fig. S19*A* shows, the performance distributions of these three tenure levels were similar; we therefore combined them into a common category labeled “physicians.” An additional 11,772 diagnoses were contributed by 1,037 medical students; on average, these were less accurate (*SI Appendix*, Fig. S19 *A* and *B*).

For hybrid human–LLM ensembles (Fig. 3), only cases diagnosed by a minimum of five physicians were analyzed (so that collectives of up to five humans could be simulated), totaling 1,928 cases. The gold-standard solution for each case, considered the correct diagnosis in this analysis, consists of one or more diagnoses provided by case authors. These diagnoses are then vetted by the Human Dx editorial board, a team of licensed medical professionals, to confirm that the clinical information in each vignette is sufficient to support the correct diagnosis and that each case provides a positive learning experience. Notably, the gold-standard solution is established before any user attempts to diagnose the case on the Human Dx platform. A current list of contributing editors who author and review clinical cases is available at: www.humandx.org/editors. We use this gold-standard solution as the basis for calculating accuracy metrics in our analyses. The medical specialty of a case (used for the robustness analyses reported in *SI Appendix*, Figs. S5 and S14) was determined by prompting Anthropic Claude 3 Opus to identify the three most probable specialties from a list of 145 specialties used internally by Human Dx (see *SI Appendix* for the exact wording of the prompt). Only the most probable specialty was used in the analyses shown in *SI Appendix*, Figs. S5 and S14.

### 9.2. LLMs: Prompt Engineering and Postprocessing of Responses.

Prompt engineering can markedly affect the quality and format of LLM responses. There is no established framework for prompt engineering, and which wording produces the desired response typically depends on the LLM used. Some studies have found that shorter prompts work better (88); others that complex prompts yield better responses (89). In practice, prompts are generally engineered by trial and error (89).

We took a systematic, semi-exhaustive approach, building up prompts in a modular fashion by concatenating several text blocks (*SI Appendix*, Fig. S1). The most basic block feeds the case vignette to the LLM verbatim. The case vignette describes the patient’s symptoms, test results, and medical record. The LLM is then asked to provide the five most probable diagnoses ordered by their likelihood of being correct (i.e., a differential diagnosis). We included several additional text blocks in the prompt and tested whether these additions increased diagnostic accuracy. The prompt that performed best in a training fold of cases was then used for the analysis in the remaining folds. Specifically, the additional text blocks assign the LLM the role of a medical expert [impersonation (90)], advise it to check that the proposed diagnoses are consistent with the case description (self-consistency), advise the LLM to report diagnoses in SNOMED CT terminology (answer format SCT) or in common shorthand (answer format common), or offer five examples of case vignettes with their correct diagnoses [a technique known as few-shot prompting; (91)]. In selecting the few-shot examples, we sought to ensure variety in patients’ age (5 mo to 89 y) and gender (3 female and 2 male) and the medical specialty. The resulting LLM responses constitute the basis of the results reported here. For details of the exact wording of prompts and results of the validation process, see *SI Appendix*, Fig. S1.

Our general validation approach is as follows: We used 10-times repeated five-fold cross-validation on the whole set of cases, using onefold of cases to select the best prompt and calculate the weights for humans and LLMs (*Weighted Aggregation of LLMs and/or Human Inputs*). The other four folds were used for assessing out-of-sample performance. We report results averaged across the fifty cross-validation outcomes.

The raw LLM responses required some additional postprocessing (which was not needed for the human responses). Even when explicitly instructed to provide answers in a specific format, some LLMs did not always comply and occasionally returned verbose responses. However, these responses follow typical patterns that are easy to recognize. Some LLMs, for example, start the response with an introductory sentence before parsing the differential diagnosis in the requested format. We therefore removed the response until the first line break if the response started with “Sure,...,” “Here is the...,” “Here are...,” “### Response:...,” “The probable...,” “The differential...,” “The most probable...,” or “Based on...”. Furthermore, we removed various forms of list numbering.

### 9.3. Matching Raw Text to Unique Medical Concepts (SNOMED CT).

One of the main challenges when aggregating individual diagnoses in open-ended medical diagnostics is discerning which diagnoses correspond to the same medical concept. The differential diagnoses given by humans and LLMs consist of raw text. Two strings pointing to the same disease might differ slightly—for example, due to typos, use of synonyms, or differences in spelling. To facilitate comparison of these open-ended diagnoses, we developed a method and processing pipeline that leveraged the comprehensive SNOMED CT healthcare terminology (March 2023 International Edition Release) and mapped the raw string responses to unique IDs in SNOMED CT (extending a pipeline described in ref. 34).

The first step is string normalization, using routine natural language processing tools to standardize all diagnoses—including the correct ones provided by cases’ authors. The normalization procedure involves removing stop words, converting British English to US English, converting plural to singular, and identifying acronyms; specifically, we used the Norm^*^ pipeline, one of the Lexical Tools maintained by the National Library of Medicine. The second step is to map concepts to SNOMED CT IDs (Fig. 1*C*). This is done by comparing a normalized diagnosis string to the normalized entries in SNOMED CT including all of their stored synonyms sharing the same ID. A SNOMED CT ID is assigned to a diagnosis only when there is an exact match between the sets of words—in other words, the compared strings having a Jaccard similarity of 1. On the rare occasion that more than one SNOMED CT ID is matched by this technique, SNOMED CT allows for differentiation by semantic tags. We gave preference to SNOMED IDs according to their semantic tags in the following order: “disorder,” “finding,” “morphologic abnormality,” “body structure,” “person,” “organism,” “specimen” (see ref. 34 for the rationale behind this ordering), so that a diagnosis was only matched to exactly one ID.

Applying this approach, as described in ref. 34, produced a match for 90% of the correct case diagnoses, 78% of diagnoses given by LLMs (calculated across all prompts), and 84% of diagnoses given by humans. For the diagnoses that could not be matched, we employed a different approach. We created 768-dimensional vector embeddings of all unique (active) SNOMED CT concepts and synonyms using a sentence-transformer model based on the *pubmedbert* model (92)—a domain-specific transformer model trained on texts from the *National Library of Medicine* and fine-tuned over the *MS-MARCO* dataset using the sentence-transformer framework (93). We then created a vector embedding of the diagnosis to be matched and assigned it the SNOMED CT ID for which the cosine similarity between embedding vectors was highest. We were thus able to match all remaining raw string diagnoses to exactly one SNOMED CT ID. For example, the diagnosis “Chlamydia infection” which could not be matched before was now correctly matched to the SNOMED CT concept “Chlamydial infection (disorder).” Likewise, “HIV disease” was correctly matched to the SNOMED CT concept “HIV infection (disorder).” As a sanity check, we applied the sentence-transformer matching technique to all diagnoses that were successfully matched in the first approach (i.e., using the pipeline described in ref. 34) and found that both methods arrived at the same SNOMED CT ID for 99.4% of diagnosis strings (given by humans or LLMs).

Applying this mapping approach allowed us to systematically quantify the number and diversity of diagnoses in the dataset. The 2,133 medical cases contained a total of 2,008 unique diagnosis strings provided by case authors as correct solutions, which were mapped to 1,610 unique SNOMED CT concepts for standardization. Across all responses, humans and LLMs together generated 63,732 unique diagnosis strings, with 14,448 from humans and 53,454 from LLMs (including responses from all tested prompts). These were mapped to 18,130 unique SNOMED CT concepts, of which 9,218 were provided by humans and 14,251 by LLMs.

### 9.4. Weighted Aggregation of LLMs and/or Human Inputs.

To aggregate individual diagnoses into a collective diagnosis, we implemented a scoring rule. After normalizing all differential diagnoses and matching them to unique SNOMED CT IDs, we built a set of all nominated IDs (see Fig. 1*B*–*D* and previous subsection). Then, for each diagnostician (physician or LLM) and each diagnosis, a partial score was assigned that was discounted depending on the rank r in the differential diagnosis (i.e., the list of diagnoses ordered in descending order of judged probability of being the correct diagnosis). Following refs. 33 and 34, we employed a 1/r rule for the rank-discounted partial score (i.e., the inverse rank of a diagnosis). Additionally, this partial score was multiplied by a weight at the level of the diagnostician (see next paragraph). Finally, for each nominated diagnosis, these partial scores were summed up over all diagnosticians, and the ranking of the collective differential diagnosis was defined as a list sorted in decreasing order of the overall score a diagnosis received.

Prior research on CI in medical diagnostics has shown that giving equal weight to members in a collective when aggregating individual judgements into a collective diagnosis (i.e., using a simple equal-weighting combination rule) performs well as long as there is not much difference in individual performance (29). However, if there are substantial differences in individual accuracy, giving the more competent individuals higher weights in the aggregation step may improve performance. We therefore used the Weighted Majority Voting Ensemble (WMVE) approach described in ref. 94 to determine weights for LLMs and humans. Weights were determined on one-fifth of the cases and calculated for each configuration (i.e., combinations for the accuracy metric used and which LLMs and/or the number of human experts). The performance of the WMVE was then calculated on the remaining four-fifths of the cases. Results are reported as the means of a 10-times repeated five-fold cross-validation (*LLMs: Prompt Engineering and Post-Processing of Responses*). At the start of the weight-learning process, each diagnostician j in an ensemble of n diagnosticians (physicians or LLMs) is assigned a weight of $w_{j,0}=1$. For each case i in the training set, the weights are updated according to $w_{j,i}=w_{j,i-1}+\alpha_{i}$, where $\alpha_{i}=s_{j,i}\cdot \left(n-\sum_{j=1}^{n}s_{j,i}\right)/n$ and $s_{j,i}$ is the score of diagnostician j achieved on case i, which depends on the performance metric used (for top-k, it is either 1 or 0; for reciprocal rank, it is 1/r); that is, we estimated weights separately for each metric we evaluated. This means that the weight increases if a diagnostician correctly diagnoses a case in the training set, with a larger increase if the diagnoses of other diagnosticians in the ensemble are incorrect. It was not possible to calculate a weight for each individual physician because many only rated a few (or none) of the cases in the training set. We therefore calculated a shared, average weight for all physicians. To this end, for each case in the training set and for each hybrid configuration with n humans, we built all possible groups of n physicians (i.e., using the physicians who provided a differential diagnosis for that case) and averaged over them. If the number of possible groups exceeded 100, we randomly sampled 100 unique groups. In most cases, applying such a weighted combination rule outperformed a simple equal-weighting combination rule. However, even with equal weights applied, LLM and hybrid ensembles generally outperformed individual LLMs and physicians (*SI Appendix*, Fig. S20). In real-world clinical applications, the feasibility of our proposed weighting method will depend on whether reliable knowledge about past performance—or at least past decision similarity (95)—is available.

## 10. Ethics Declarations

We did not collect data specifically for this study; instead, we analyzed existing data provided by Human Dx. When users sign up on the Human Dx platform, they give consent for their data to be processed and analyzed for research purposes. We consulted the Ethics Committee of the Max Planck Institute for Human Development, which deemed our study exempt from approval. We have complied with all relevant ethical regulations regarding data protection.

## Supplementary Material

Appendix 01 (PDF)

## Data Availability

Human Dx commits to providing access to the entire dataset needed to reproduce the analyses presented upon request by any researcher employed at an accredited academic institution, for the sole purpose of independently verifying and reproducing the results presented in this manuscript. This controlled data access protocol is in place due to privacy considerations associated with vignettes representing patients seen in clinical practice and performance data of physicians providing diagnoses for those vignettes and to safeguard against benchmark contamination for evaluating AI and human–AI collaborative systems (refs. 96 and 97). Thus, researchers must agree to use the dataset solely for validation purposes (excluding model training, commercial use, or inputting data into commercial language models that use input for training purposes). Requests for the entire dataset can be issued via the following link: www.humandx.org/data. We also include one Human Dx case along with the differential diagnoses provided by humans and LLMs to illustrate our approach, accessible at: https://github.com/nikozoe/human_ai_collectives.

## Footnotes

^*^https://lhncbc.nlm.nih.gov/LSG/Projects/lvg/current/docs/userDoc/tools/norm.html.
