# Supplementary material for: Human–AI collectives most accurately diagnose clinical vignettes
Source: Proc Natl Acad Sci U S A. 2025 Jun 13;122(24):e2426153122. doi: 10.1073/pnas.2426153122 (PMC12184336; doi:10.1073/pnas.2426153122)
Supplement: Supplementary file 1 — Appendix 01 (PDF) [file pnas.2426153122.sapp.pdf]

PNAS

Supporting Information for

Human-AI collectives produce the most accurate differential diagnoses

Nikolas Zöller, Julian Berger, Irving Lin, Nathan Fu, Jayanth Komarneni, Gioele Barabucci, Kyle Laskowski, Victor Shia, Benjamin Harack, Eugene A. Chu, Vito Trianni, Ralf H.J.M. Kurvers and Stefan M. Herzog

Nikolas Zöller
E-mail: zoeller@mpib-berlin.mpg.de

This PDF file includes:

Supporting text
Figs. S1 to S20
Table S1
SI References

Supporting Information Text

A. Example case vignette.

40 year old female presenting with chest pain
Symptom: Worsening chest pain

- Onset: 2 weeks ago
- Associated with: Cough, dyspnea, fever
- Complicated by: Fatigue

Social history

- Recent construction in Ohio

Physical exam

- Lungs: Wheezing

Diagnostic: X-ray

- Interpretation: Normal

B. Verbatim text of LLM prompts. Below we list the text of the building blocks used to construct prompts for eliciting differential diagnoses. For all LLMs, we tested all feasible combinations of these building blocks. Diagnostic accuracies for each LLM and each prompt combination on the whole dataset of 2133 cases are presented in SI Table S1.
Base task: *Provide only the most probable differential diagnosis, no explanation, no recapitulation of the case information or task. Give a maximum of 5 answers, sorted by probability of being the correct diagnosis, most probable first, remove list numbering, and respond with each answer on a new line. Be as concise as possible, no need to be polite.*
Impersonation: *You are a medical expert diagnosing a patient.*
Response format: common shorthand: *In your answer use common shorthand non-abbreviated diagnoses.*
Response format: SCT: *In your answer provide only the appropriate SNOMED CT fully specified name, no id.*
Self-consistency: *Check that each differential diagnosis in your answer is consistent with each finding in the case description.*
Few-shot prompt: *Here are some examples of cases and their correct answers:*
Case description: {case vignette}
Answer: {example solution} (5x)

The prompt used to have Anthropic Claude 3 Opus assign a medical specialty to a case vignette was as follows:
In the list of medical specialties within the following <SP>\<SP>tags <SP>{specialties_one_line}<\SP>, each specialty is separated by a comma. You are given the following case in the <case>\<case>tags: <case>{case_text}<\case>. Follow the steps provided: 1. Determine the top 3 choices for which specialties best fit the case. 2. Give your answer in an ordered numbered list starting with the most confident answer first. Only answer with the list. Do not provide any additional explanation.

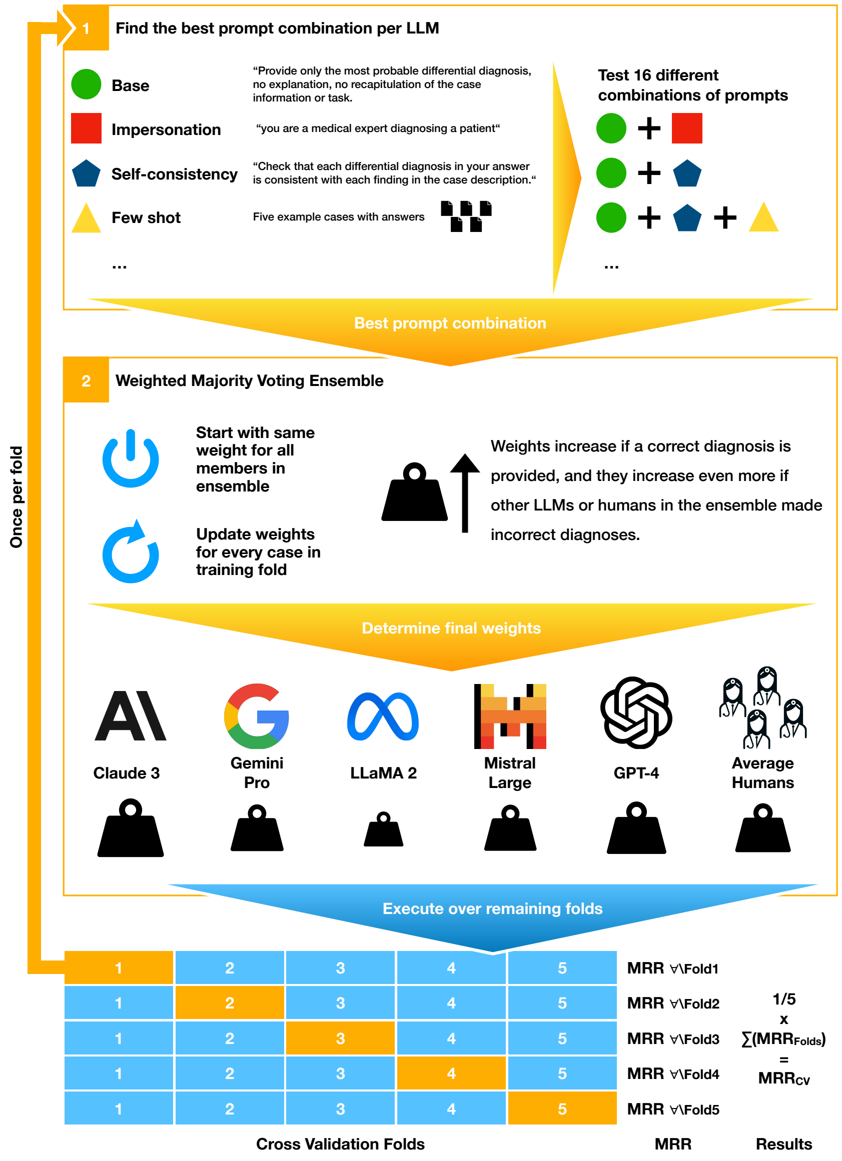

Fig. S1. Illustration of LLM prompt engineering and validation method. We nested our prompt engineering and Weighted Majority Voting Ensemble (WMVE) (1) sequence in a repeated five-fold cross-validation procedure. First, we determined which prompt performed best for each LLM in the training fold (one-fifth of the data; see Methods). Second, we calculated weights for each member of the ensemble, also using the training fold. The weights were then used to aggregate collective diagnoses and evaluate the ensemble's performance on the remaining folds (four-fifths of the data). This process yields one result per fold, of which the averages are reported in the main text. We repeated this procedure for every metric reported in the main text (i.e., top-1, top-3, top-5 and MRR).

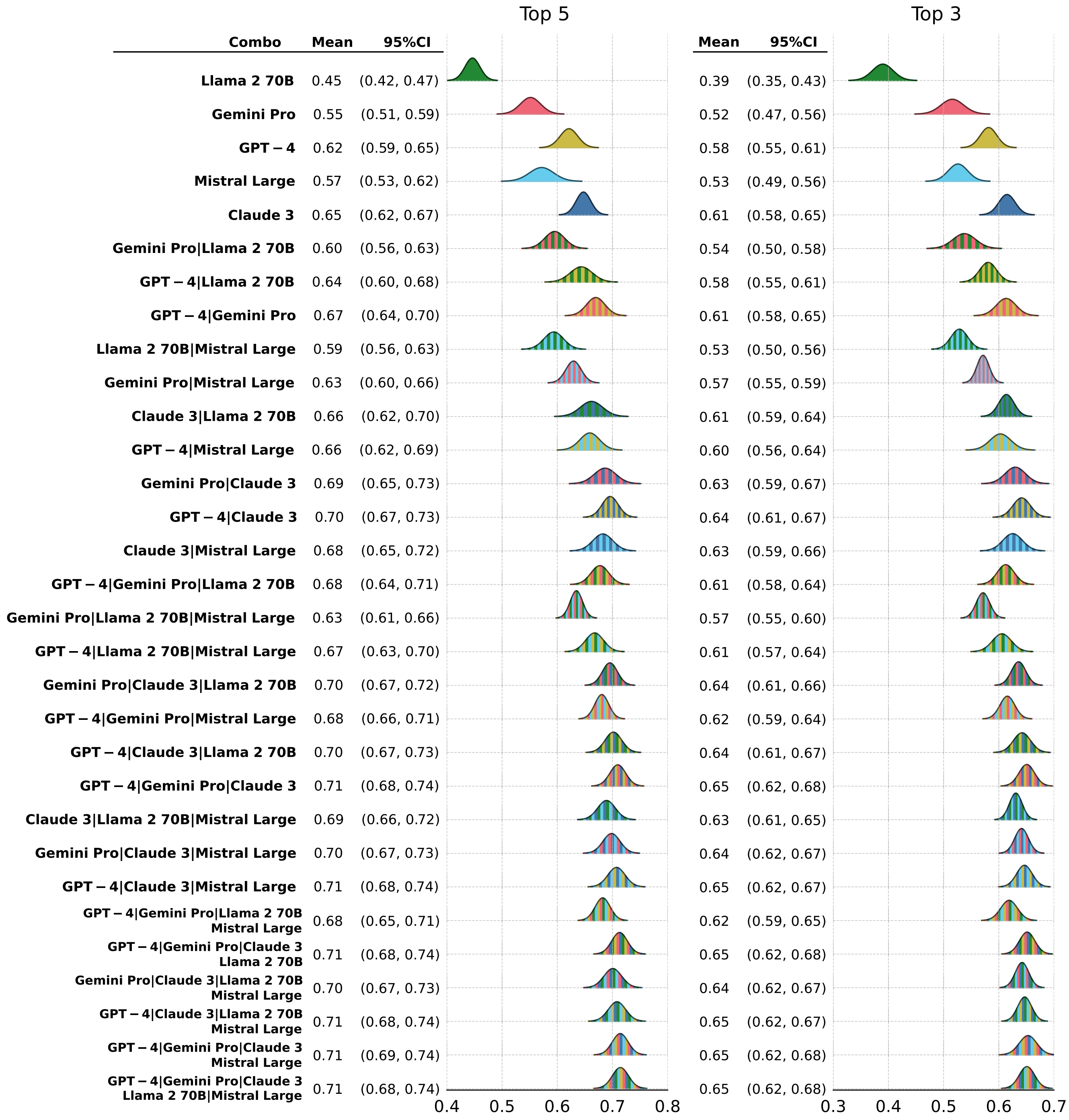

Fig. S2. Bayesian posterior distributions of cross-validated performance of five individual LLMs and ensembles of all possible combinations of LLMs. Posteriors are modeled as Student-t distributions based on 10-times repeated 5-fold cross-validation results, using (2) and applying the Nadeau-Bengio heuristic correction (3) to account for data overlap. After each label, the respective posterior is summarized using the posterior mean and a 95% equal-tailed credible interval.

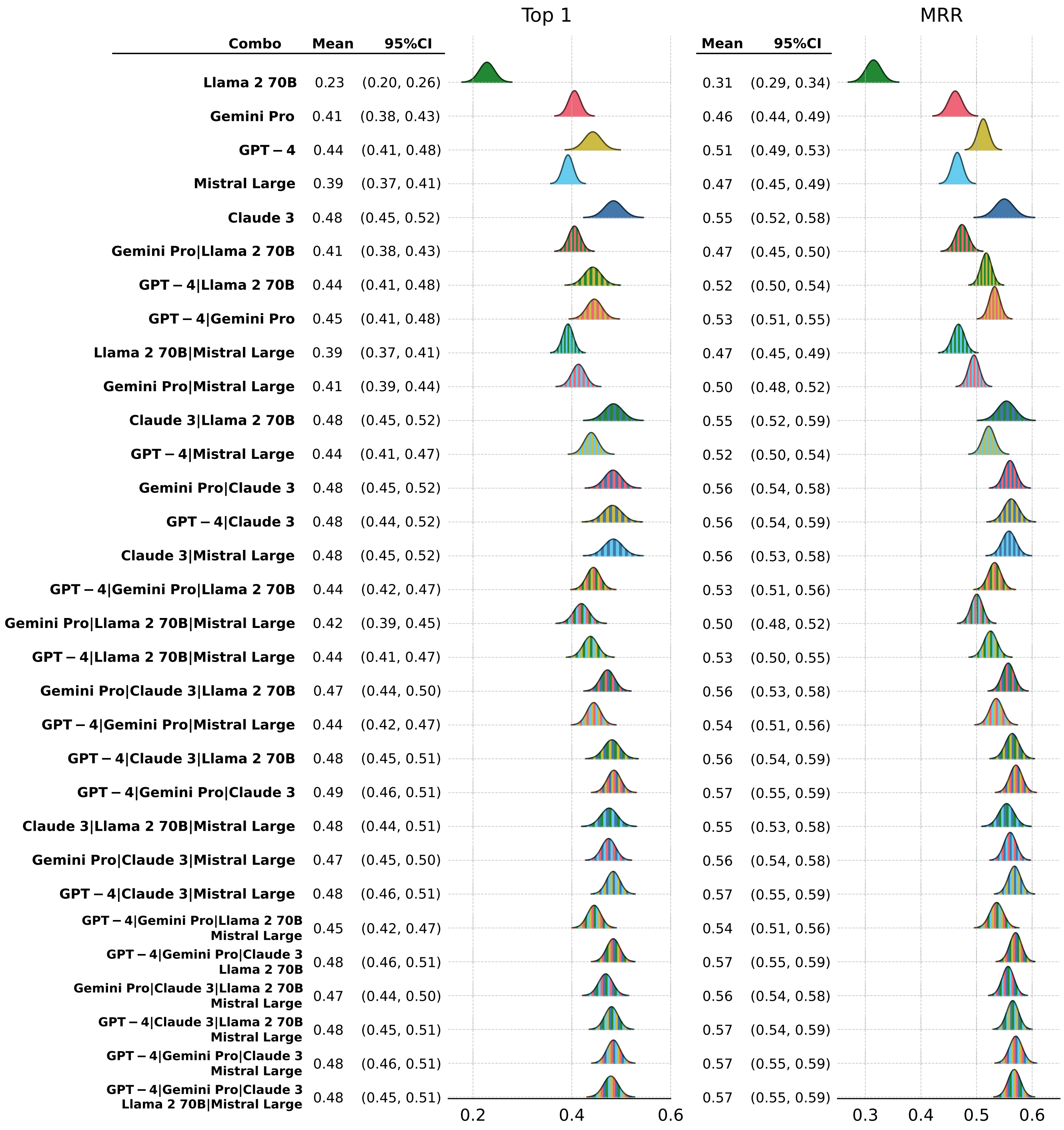

Fig. S3. Posteriors of cross-validated performance in terms of top 1 accuracy and MRR of five individual LLMs and ensembles of all possible combinations of LLMs. Posteriors are modeled as Student-t distributions based on 10-times repeated 5-fold cross-validation results, using (2) and applying the Nadeau-Bengio heuristic correction (3) to account for data overlap. After each label, the respective posterior is summarized using the posterior mean and a 95% equal-tailed credible interval.

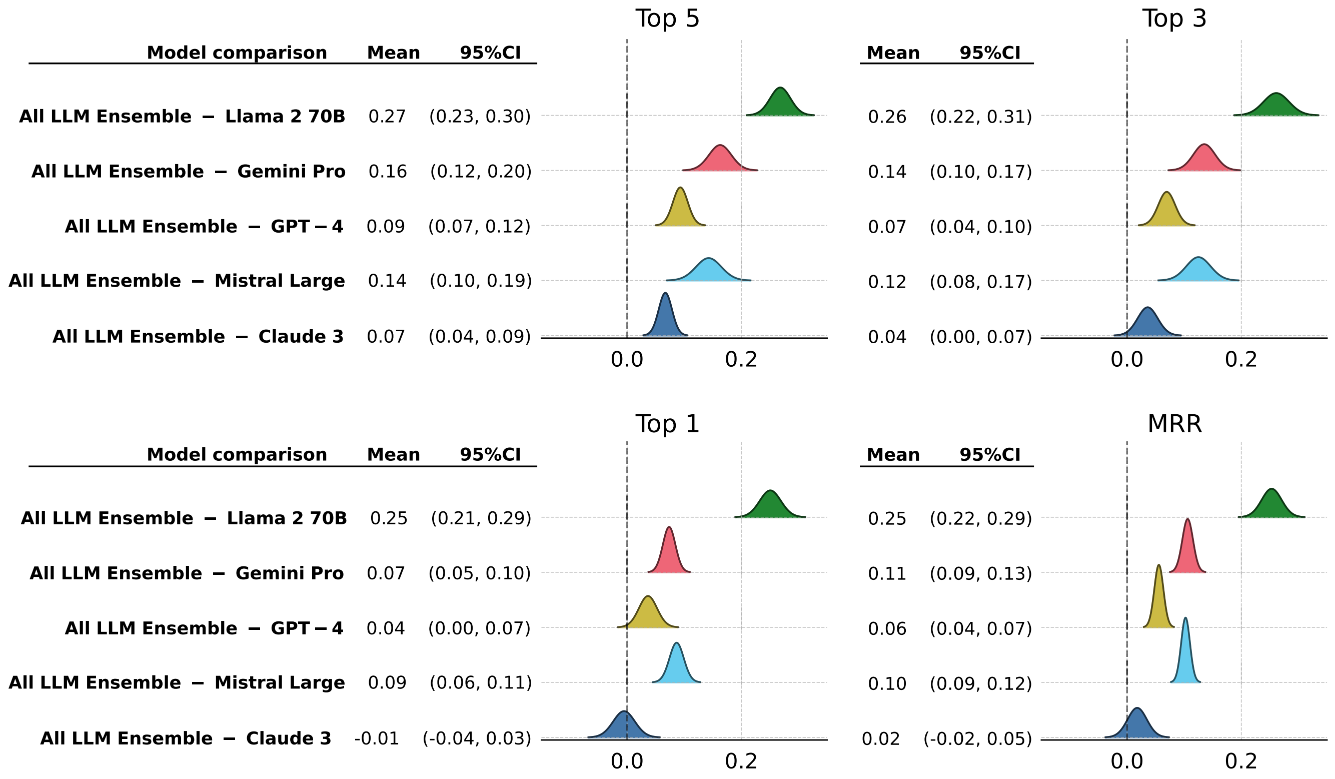

Fig. S4. Posteriors of cross-validated performance difference between an all-LLM ensemble and five individual LLMs. Posteriors are modeled as Student-t distributions based on 10-times repeated 5-fold cross-validation results, using (2) and applying the Nadeau-Bengio heuristic correction (3) to account for data overlap. After each label, the respective posterior is summarized using the posterior mean and a 95% equal-tailed credible interval. An all-LLM ensemble consistently outperforms individual LLMs across metrics, except for comparisons with Claude 3 on top-1 accuracy and MRR where differences are not statistically reliable.

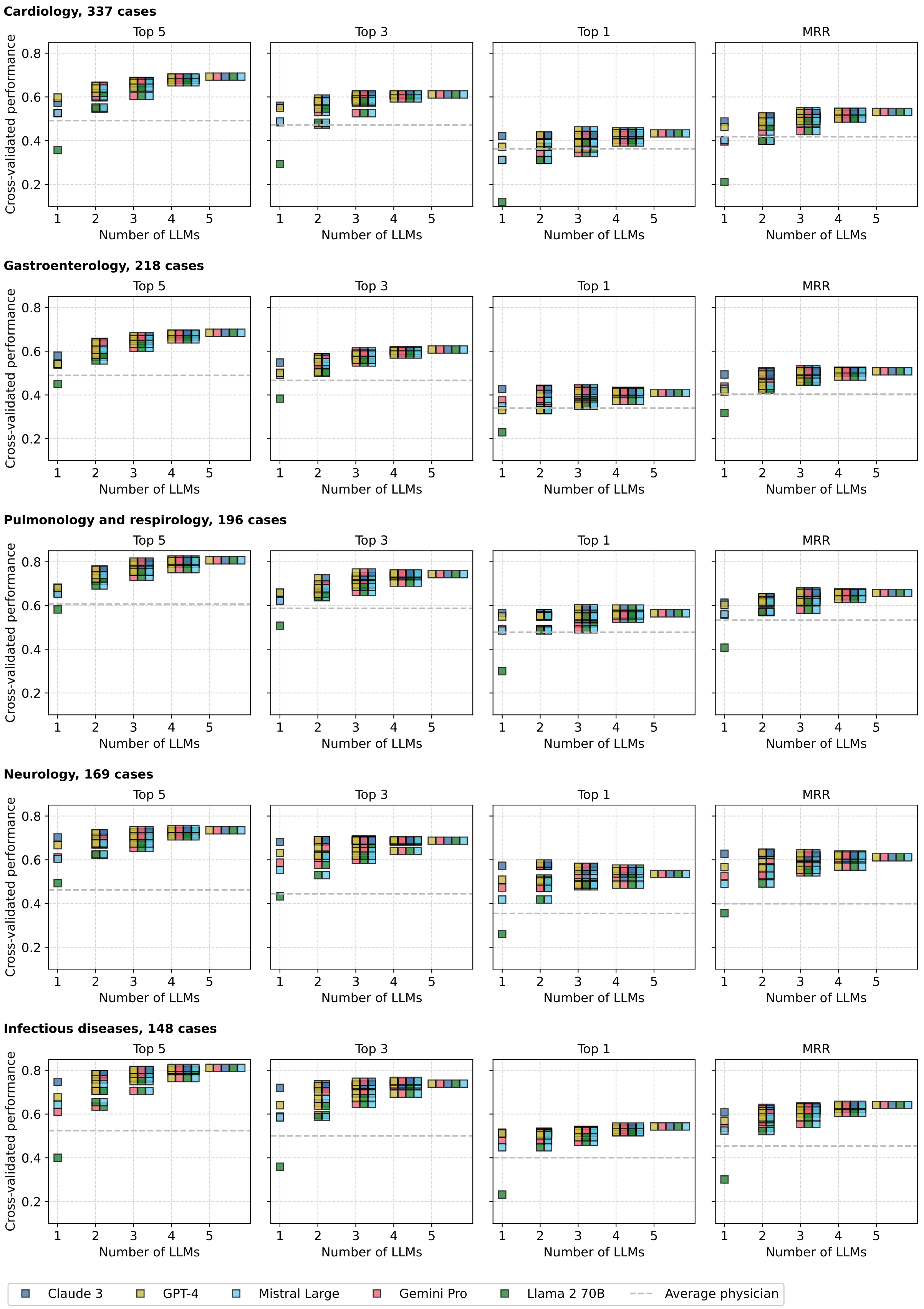

Fig. S5. Cross-validated performance of five individual LLMs and ensembles of all possible combinations of LLMs for the five most common specialties in the dataset. Across all medical specialties, combining several LLMs into a collective increased diagnostic accuracy relative to the best-performing individual LLM across all performance metrics except top-1. In most cases, the best results were obtained by combining all LLMs.

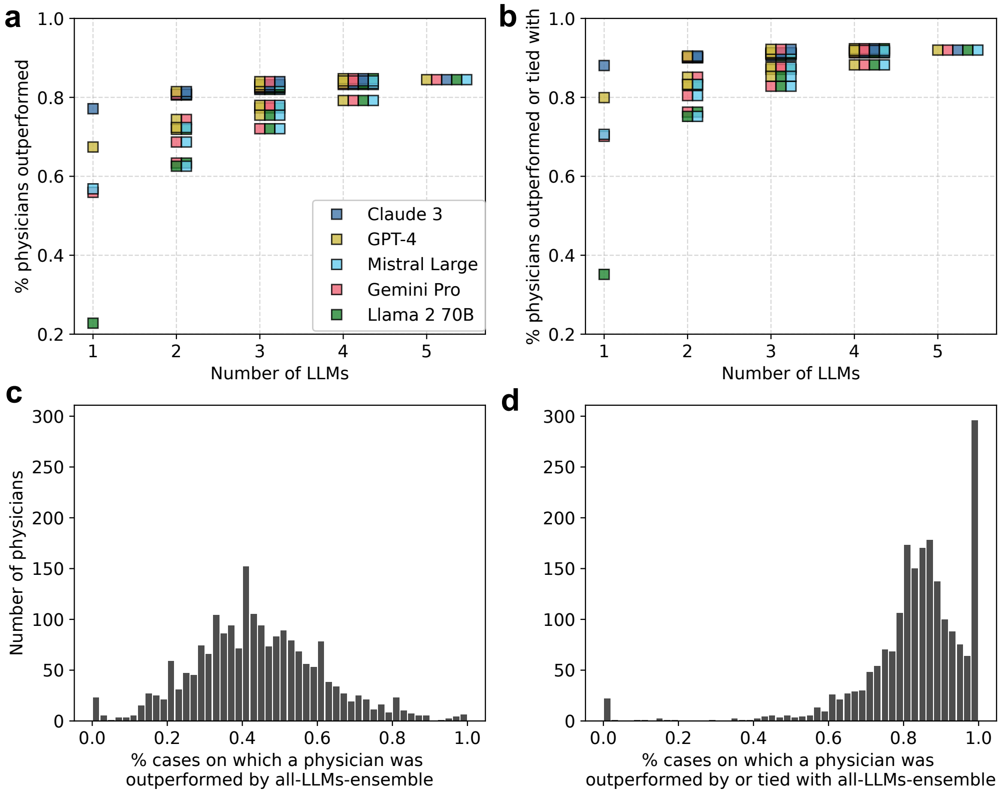

Fig. S6. Cross-validated relative performance of individual humans and LLMs. a, Percentage of physicians outperformed by an LLM across the cases they solved. The analysis was limited to physicians who diagnosed five or more cases (n = 1,997). A physician was outperformed on a case if the LLM (ensemble) placed the correct diagnosis at a higher rank; a physician was counted as outperformed overall if they were outperformed more often than they outperformed the LLM (ensemble) across their set of solved cases. **b**, Percentage of physicians outperformed by or tied with an LLM (ensemble) across the cases they solved. **c** and **d**, Results for the LLM ensemble only. **c**, Histogram of the number of physicians outperformed on a certain percentage of cases by the LLM ensemble. **d**, Histogram of the number of physicians outperformed or tied with on a certain percentage of cases by the LLM ensemble. **c** and **d** differ considerably due to the significant number of ties on the case level.

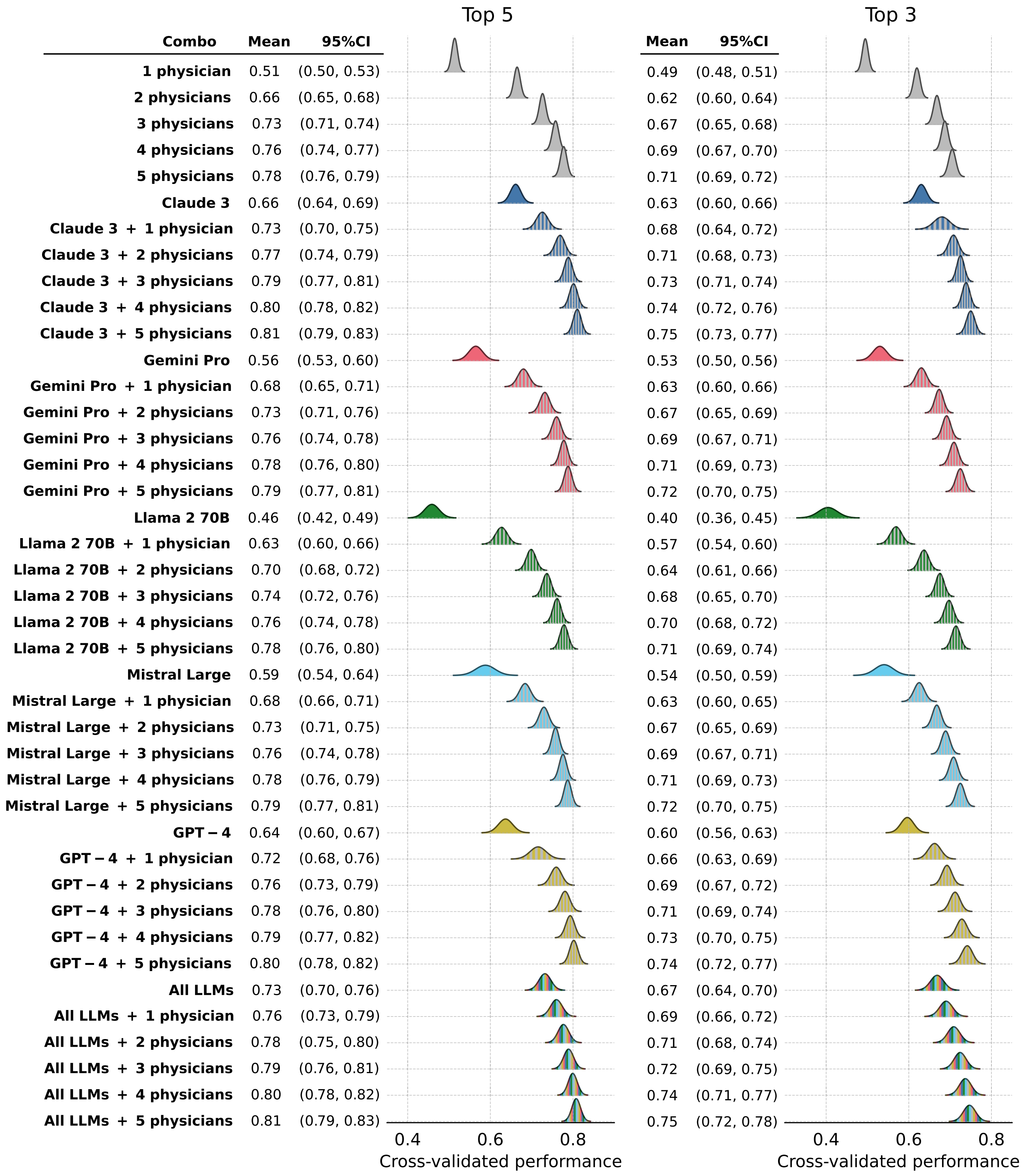

Combo	Top 5 Mean	Top 5 95%CI	Top 3 Mean	Top 3 95%CI
**1 physician**	0.51	(0.50, 0.53)	0.49	(0.48, 0.51)
**2 physicians**	0.66	(0.65, 0.68)	0.62	(0.60, 0.64)
**3 physicians**	0.73	(0.71, 0.74)	0.67	(0.65, 0.68)
**4 physicians**	0.76	(0.74, 0.77)	0.69	(0.67, 0.70)
**5 physicians**	0.78	(0.76, 0.79)	0.71	(0.69, 0.72)
**Claude 3**	0.66	(0.64, 0.69)	0.63	(0.60, 0.66)
**Claude 3 + 1 physician**	0.73	(0.70, 0.75)	0.68	(0.64, 0.72)
**Claude 3 + 2 physicians**	0.77	(0.74, 0.79)	0.71	(0.68, 0.73)
**Claude 3 + 3 physicians**	0.79	(0.77, 0.81)	0.73	(0.71, 0.74)
**Claude 3 + 4 physicians**	0.80	(0.78, 0.82)	0.74	(0.72, 0.76)
**Claude 3 + 5 physicians**	0.81	(0.79, 0.83)	0.75	(0.73, 0.77)
**Gemini Pro**	0.56	(0.53, 0.60)	0.53	(0.50, 0.56)
**Gemini Pro + 1 physician**	0.68	(0.65, 0.71)	0.63	(0.60, 0.66)
**Gemini Pro + 2 physicians**	0.73	(0.71, 0.76)	0.67	(0.65, 0.69)
**Gemini Pro + 3 physicians**	0.76	(0.74, 0.78)	0.69	(0.67, 0.71)
**Gemini Pro + 4 physicians**	0.78	(0.76, 0.80)	0.71	(0.69, 0.73)
**Gemini Pro + 5 physicians**	0.79	(0.77, 0.81)	0.72	(0.70, 0.75)
**Llama 2 70B**	0.46	(0.42, 0.49)	0.40	(0.36, 0.45)
**Llama 2 70B + 1 physician**	0.63	(0.60, 0.66)	0.57	(0.54, 0.60)
**Llama 2 70B + 2 physicians**	0.70	(0.68, 0.72)	0.64	(0.61, 0.66)
**Llama 2 70B + 3 physicians**	0.74	(0.72, 0.76)	0.68	(0.65, 0.70)
**Llama 2 70B + 4 physicians**	0.76	(0.74, 0.78)	0.70	(0.68, 0.72)
**Llama 2 70B + 5 physicians**	0.78	(0.76, 0.80)	0.71	(0.69, 0.74)
**Mistral Large**	0.59	(0.54, 0.64)	0.54	(0.50, 0.59)
**Mistral Large + 1 physician**	0.68	(0.66, 0.71)	0.63	(0.60, 0.65)
**Mistral Large + 2 physicians**	0.73	(0.71, 0.75)	0.67	(0.65, 0.69)
**Mistral Large + 3 physicians**	0.76	(0.74, 0.78)	0.69	(0.67, 0.71)
**Mistral Large + 4 physicians**	0.78	(0.76, 0.79)	0.71	(0.69, 0.73)
**Mistral Large + 5 physicians**	0.79	(0.77, 0.81)	0.72	(0.70, 0.75)
**GPT − 4**	0.64	(0.60, 0.67)	0.60	(0.56, 0.63)
**GPT − 4 + 1 physician**	0.72	(0.68, 0.76)	0.66	(0.63, 0.69)
**GPT − 4 + 2 physicians**	0.76	(0.73, 0.79)	0.69	(0.67, 0.72)
**GPT − 4 + 3 physicians**	0.78	(0.76, 0.80)	0.71	(0.69, 0.74)
**GPT − 4 + 4 physicians**	0.79	(0.77, 0.82)	0.73	(0.70, 0.75)
**GPT − 4 + 5 physicians**	0.80	(0.78, 0.82)	0.74	(0.72, 0.77)
**All LLMs**	0.73	(0.70, 0.76)	0.67	(0.64, 0.70)
**All LLMs + 1 physician**	0.76	(0.73, 0.79)	0.69	(0.66, 0.72)
**All LLMs + 2 physicians**	0.78	(0.75, 0.80)	0.71	(0.68, 0.74)
**All LLMs + 3 physicians**	0.79	(0.76, 0.81)	0.72	(0.69, 0.75)
**All LLMs + 4 physicians**	0.80	(0.78, 0.82)	0.74	(0.71, 0.77)
**All LLMs + 5 physicians**	0.81	(0.79, 0.83)	0.75	(0.72, 0.78)

Fig. S7. Bayesian posteriors of cross-validated performance for physician groups and hybrid collectives combining LLMs and physicians. Posteriors of top-5 and top-3 accuracy are modeled as Student-t distributions based on 10-times repeated 5-fold cross-validation results, using (2) and applying the Nadeau-Bengio heuristic correction (3) to account for data overlap. After each label, the respective posterior is summarized using the posterior mean and a 95% equal-tailed credible interval.

Combo	Top 1 Mean	Top 1 95%CI	MMR Mean	MMR 95%CI
**1 physician**	0.39	(0.37, 0.40)	0.44	(0.43, 0.45)
**2 physicians**	0.41	(0.40, 0.43)	0.52	(0.50, 0.53)
**3 physicians**	0.47	(0.45, 0.49)	0.57	(0.56, 0.59)
**4 physicians**	0.51	(0.49, 0.53)	0.60	(0.59, 0.62)
**5 physicians**	0.53	(0.51, 0.55)	0.63	(0.61, 0.64)
**Claude 3**	0.49	(0.45, 0.54)	0.56	(0.53, 0.59)
**Claude 3 + 1 physician**	0.50	(0.45, 0.54)	0.59	(0.55, 0.62)
**Claude 3 + 2 physicians**	0.52	(0.50, 0.55)	0.62	(0.60, 0.64)
**Claude 3 + 3 physicians**	0.54	(0.52, 0.56)	0.64	(0.62, 0.66)
**Claude 3 + 4 physicians**	0.55	(0.53, 0.58)	0.65	(0.63, 0.67)
**Claude 3 + 5 physicians**	0.56	(0.54, 0.59)	0.66	(0.64, 0.68)
**Gemini Pro**	0.42	(0.39, 0.44)	0.47	(0.44, 0.50)
**Gemini Pro + 1 physician**	0.43	(0.40, 0.46)	0.53	(0.50, 0.56)
**Gemini Pro + 2 physicians**	0.48	(0.45, 0.51)	0.58	(0.55, 0.60)
**Gemini Pro + 3 physicians**	0.51	(0.49, 0.54)	0.61	(0.58, 0.63)
**Gemini Pro + 4 physicians**	0.53	(0.51, 0.56)	0.63	(0.60, 0.65)
**Gemini Pro + 5 physicians**	0.55	(0.52, 0.57)	0.64	(0.62, 0.66)
**Llama 2 70B**	0.24	(0.20, 0.28)	0.33	(0.30, 0.36)
**Llama 2 70B + 1 physician**	0.39	(0.37, 0.41)	0.49	(0.46, 0.51)
**Llama 2 70B + 2 physicians**	0.45	(0.42, 0.47)	0.55	(0.53, 0.57)
**Llama 2 70B + 3 physicians**	0.49	(0.47, 0.51)	0.59	(0.57, 0.61)
**Llama 2 70B + 4 physicians**	0.52	(0.50, 0.54)	0.61	(0.59, 0.63)
**Llama 2 70B + 5 physicians**	0.54	(0.52, 0.56)	0.63	(0.61, 0.65)
**Mistral Large**	0.40	(0.36, 0.44)	0.48	(0.44, 0.52)
**Mistral Large + 1 physician**	0.42	(0.39, 0.46)	0.53	(0.50, 0.56)
**Mistral Large + 2 physicians**	0.48	(0.45, 0.50)	0.58	(0.56, 0.60)
**Mistral Large + 3 physicians**	0.51	(0.49, 0.53)	0.61	(0.59, 0.63)
**Mistral Large + 4 physicians**	0.53	(0.51, 0.55)	0.63	(0.61, 0.65)
**Mistral Large + 5 physicians**	0.55	(0.53, 0.57)	0.64	(0.62, 0.66)
**GPT − 4**	0.46	(0.42, 0.49)	0.53	(0.50, 0.55)
**GPT − 4 + 1 physician**	0.46	(0.43, 0.49)	0.56	(0.54, 0.59)
**GPT − 4 + 2 physicians**	0.50	(0.48, 0.53)	0.60	(0.58, 0.62)
**GPT − 4 + 3 physicians**	0.53	(0.51, 0.55)	0.63	(0.60, 0.65)
**GPT − 4 + 4 physicians**	0.55	(0.52, 0.57)	0.64	(0.62, 0.67)
**GPT − 4 + 5 physicians**	0.56	(0.54, 0.58)	0.65	(0.63, 0.68)
**All LLMs**	0.49	(0.47, 0.52)	0.58	(0.56, 0.61)
**All LLMs + 1 physician**	0.51	(0.48, 0.54)	0.61	(0.58, 0.63)
**All LLMs + 2 physicians**	0.53	(0.50, 0.55)	0.62	(0.59, 0.65)
**All LLMs + 3 physicians**	0.54	(0.51, 0.57)	0.63	(0.60, 0.67)
**All LLMs + 4 physicians**	0.55	(0.52, 0.58)	0.65	(0.62, 0.67)
**All LLMs + 5 physicians**	0.55	(0.53, 0.58)	0.65	(0.62, 0.68)

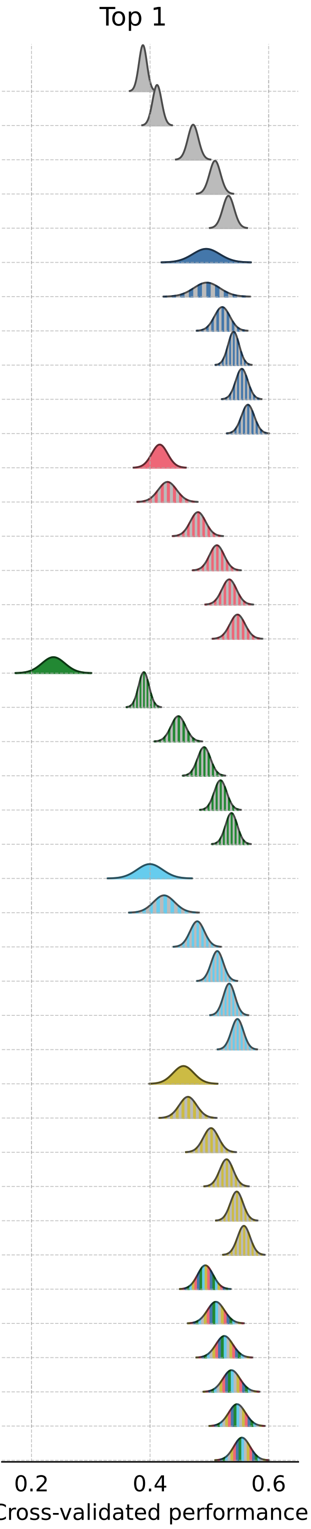

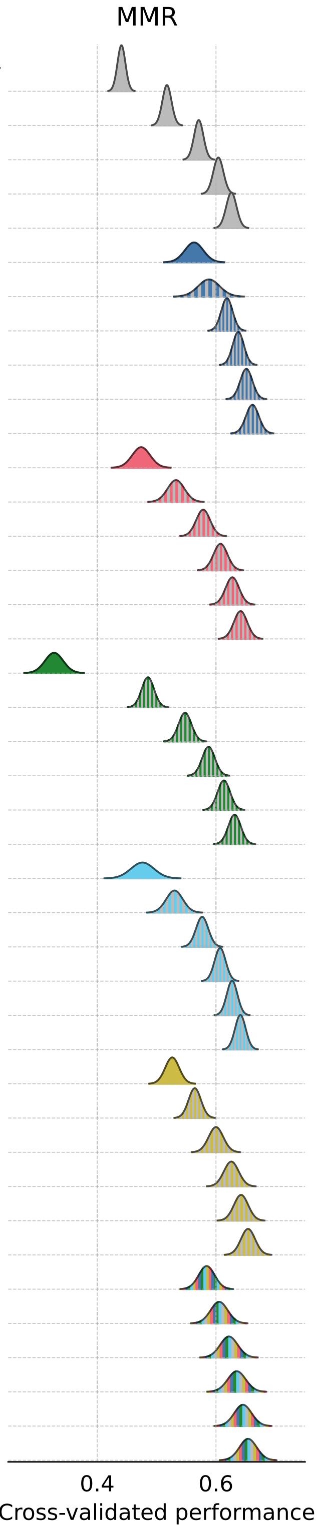

Fig. S8. Bayesian posteriors of cross-validated performance for physician groups and hybrid collectives combining LLMs and physicians. Posteriors of top-1 accuracy and MRR are modeled as Student-t distributions based on 10-times repeated 5-fold cross-validation results, using (2) and applying the Nadeau-Bengio heuristic correction (3) to account for data overlap. After each label, the respective posterior is summarized using the posterior mean and a 95% equal-tailed credible interval.

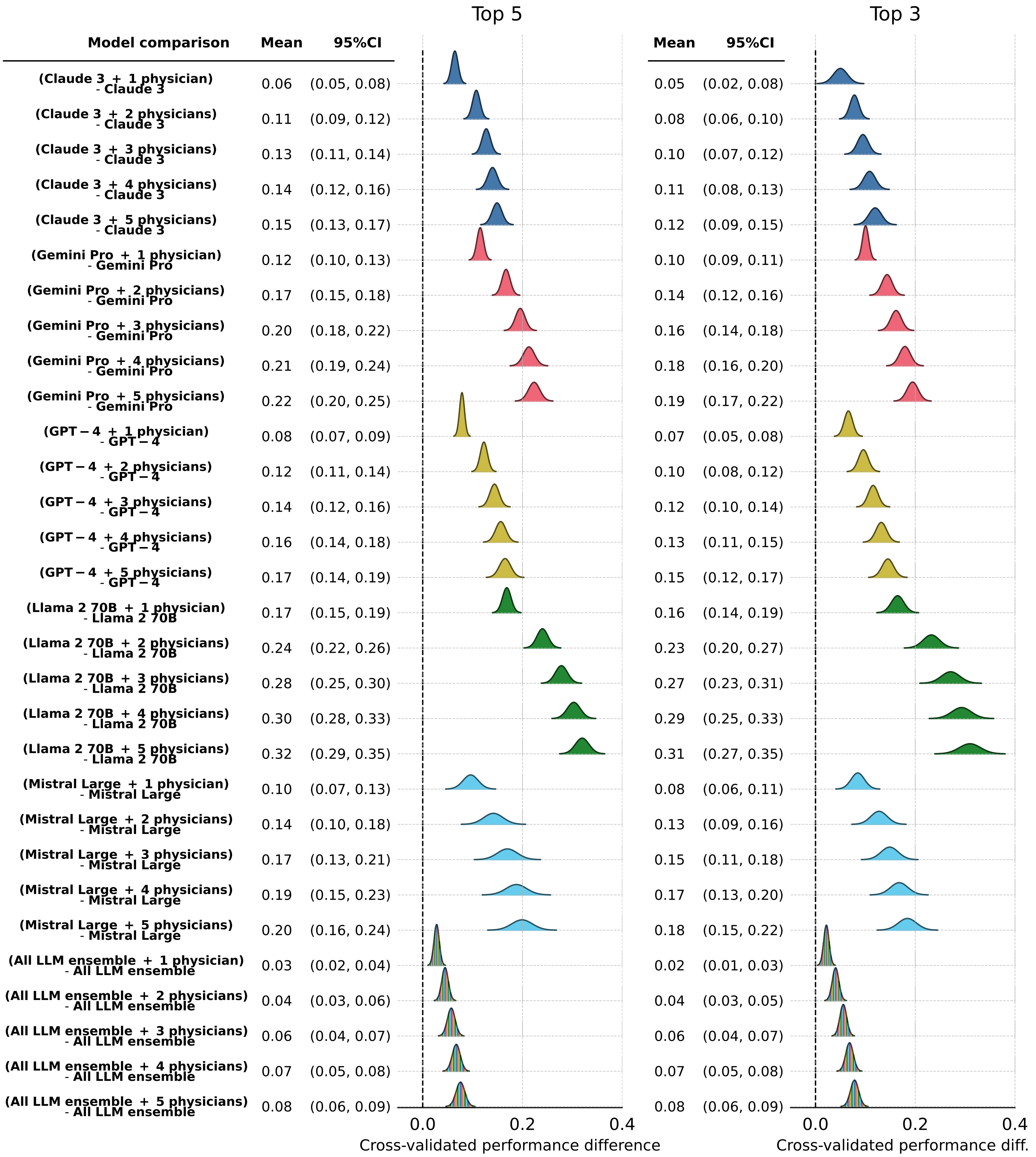

Fig. S9. Bayesian posteriors of cross-validated performance differences between hybrid ensembles and LLM ensembles/individual LLMs. Hybrid ensembles, combining LLMs with one or more physicians, consistently outperform individual LLMs and LLM-only ensembles in top-5 and top-3 accuracy. Posteriors are modeled as Student-t distributions based on 10-times repeated 5-fold cross-validation results, using (2) and applying the Nadeau-Bengio heuristic correction (3) to account for data overlap.

Model comparison	Top 1 Mean	Top 1 95%CI	MRR Mean	MRR 95%CI
(Claude 3 + 1 physician) - Claude 3	0.00	(-0.01, 0.01)	0.03	(0.01, 0.04)
(Claude 3 + 2 physicians) - Claude 3	0.03	(-0.00, 0.06)	0.06	(0.03, 0.08)
(Claude 3 + 3 physicians) - Claude 3	0.05	(0.00, 0.09)	0.07	(0.05, 0.10)
(Claude 3 + 4 physicians) - Claude 3	0.06	(0.01, 0.11)	0.09	(0.05, 0.12)
(Claude 3 + 5 physicians) - Claude 3	0.07	(0.02, 0.12)	0.10	(0.06, 0.14)
(Gemini Pro + 1 physician) - Gemini Pro	0.01	(-0.00, 0.03)	0.06	(0.05, 0.06)
(Gemini Pro + 2 physicians) - Gemini Pro	0.06	(0.05, 0.08)	0.10	(0.09, 0.12)
(Gemini Pro + 3 physicians) - Gemini Pro	0.10	(0.08, 0.12)	0.13	(0.12, 0.15)
(Gemini Pro + 4 physicians) - Gemini Pro	0.12	(0.10, 0.14)	0.15	(0.14, 0.17)
(Gemini Pro + 5 physicians) - Gemini Pro	0.13	(0.11, 0.16)	0.17	(0.15, 0.18)
(GPT − 4 + 1 physician) - GPT − 4	0.01	(-0.00, 0.02)	0.04	(0.03, 0.05)
(GPT − 4 + 2 physicians) - GPT − 4	0.05	(0.02, 0.08)	0.07	(0.05, 0.09)
(GPT − 4 + 3 physicians) - GPT − 4	0.07	(0.04, 0.11)	0.10	(0.08, 0.12)
(GPT − 4 + 4 physicians) - GPT − 4	0.09	(0.05, 0.13)	0.12	(0.09, 0.14)
(GPT − 4 + 5 physicians) - GPT − 4	0.10	(0.06, 0.14)	0.13	(0.10, 0.15)
(Llama 2 70B + 1 physician) - Llama 2 70B	0.15	(0.11, 0.19)	0.16	(0.14, 0.17)
(Llama 2 70B + 2 physicians) - Llama 2 70B	0.21	(0.18, 0.24)	0.22	(0.20, 0.24)
(Llama 2 70B + 3 physicians) - Llama 2 70B	0.25	(0.22, 0.29)	0.26	(0.24, 0.28)
(Llama 2 70B + 4 physicians) - Llama 2 70B	0.28	(0.25, 0.32)	0.29	(0.27, 0.31)
(Llama 2 70B + 5 physicians) - Llama 2 70B	0.30	(0.27, 0.33)	0.30	(0.28, 0.33)
(Mistral Large + 1 physician) - Mistral Large	0.02	(-0.01, 0.06)	0.05	(0.04, 0.07)
(Mistral Large + 2 physicians) - Mistral Large	0.08	(0.05, 0.11)	0.10	(0.08, 0.13)
(Mistral Large + 3 physicians) - Mistral Large	0.11	(0.08, 0.15)	0.13	(0.10, 0.16)
(Mistral Large + 4 physicians) - Mistral Large	0.13	(0.10, 0.17)	0.15	(0.12, 0.18)
(Mistral Large + 5 physicians) - Mistral Large	0.15	(0.11, 0.19)	0.16	(0.13, 0.20)
(All LLM ensemble + 1 physician) - All LLM ensemble	0.02	(0.00, 0.03)	0.02	(0.01, 0.03)
(All LLM ensemble + 2 physicians) - All LLM ensemble	0.03	(0.01, 0.05)	0.04	(0.03, 0.05)
(All LLM ensemble + 3 physicians) - All LLM ensemble	0.04	(0.02, 0.06)	0.05	(0.04, 0.06)
(All LLM ensemble + 4 physicians) - All LLM ensemble	0.05	(0.03, 0.07)	0.06	(0.05, 0.08)
(All LLM ensemble + 5 physicians) - All LLM ensemble	0.06	(0.04, 0.08)	0.07	(0.05, 0.09)

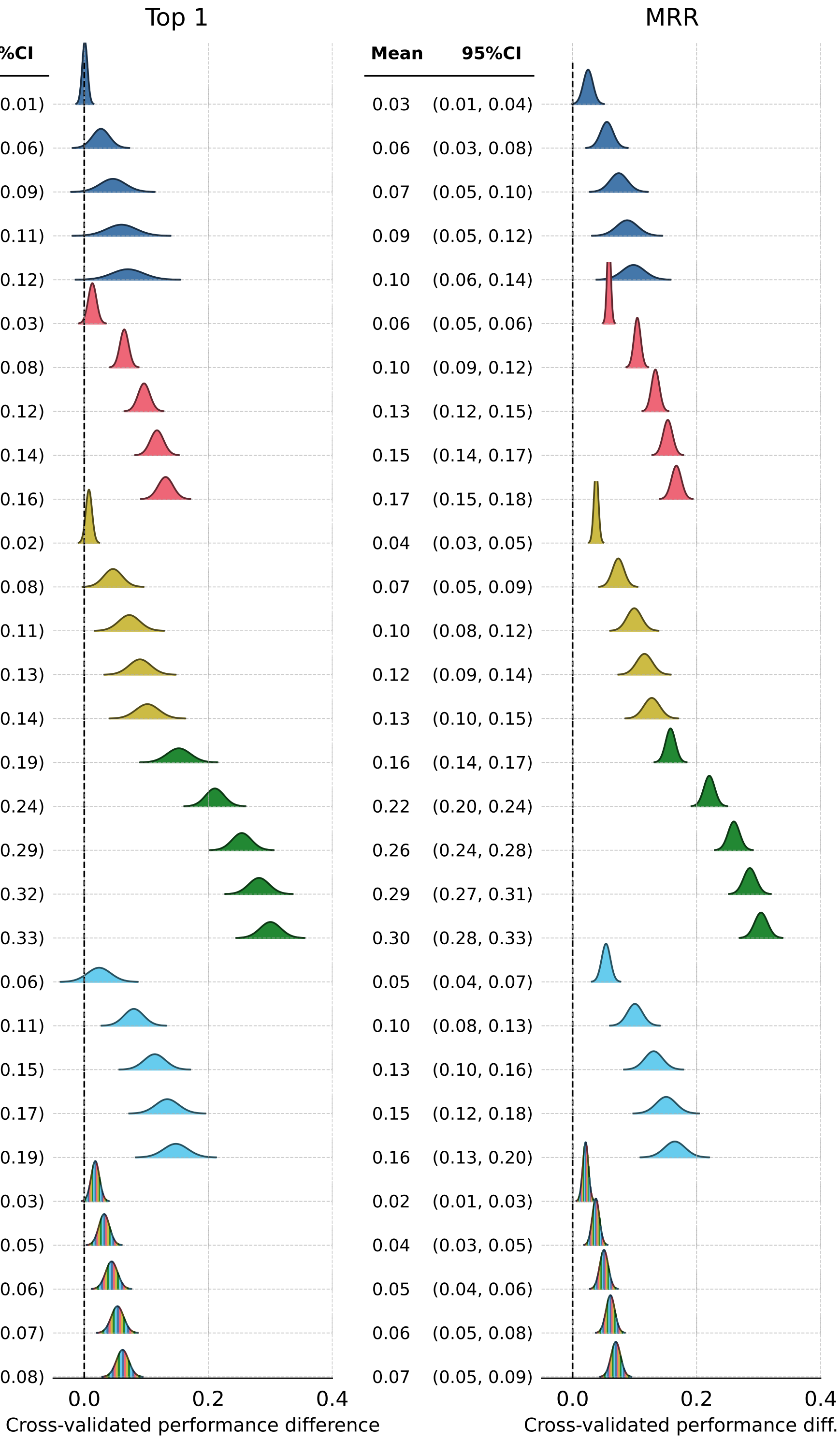

Fig. S10. Bayesian posteriors of cross-validated performance differences between hybrid ensembles and LLM ensembles/individual LLMs. Hybrid ensembles, combining LLMs with one or more physicians, consistently outperform individual LLMs and LLM-only ensembles in terms of MRR and almost always in terms of top-1 accuracy (exception: for Anthropic Claude 3 Opus and Mistral Large adding 1 physician is not significantly different). Posteriors are modeled as Student-t distributions based on 10-times repeated 5-fold cross-validation results, using (2) and applying the Nadeau-Bengio heuristic correction (3) to account for data overlap. After each label, the respective posterior is summarized using the posterior mean and a 95% equal-tailed credible interval.

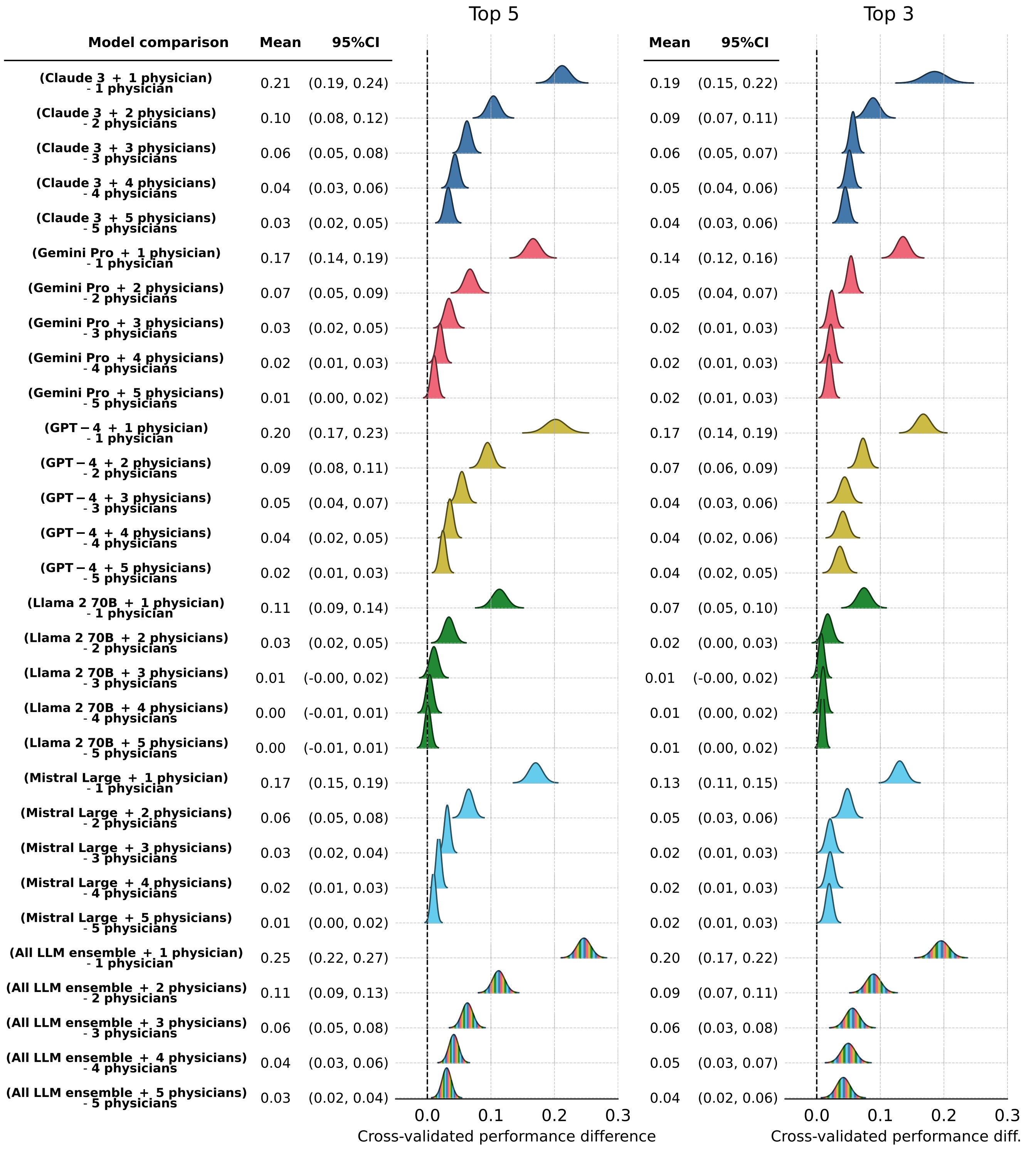

Fig. S11. Bayesian posteriors of cross-validated performance differences between hybrid ensembles and human-only ensembles. Hybrid ensembles, combining LLMs with one or more physicians, consistently outperform human-only ensembles in top-5 and top-3 accuracy. Only for groups of 4 or 5 physicians adding Meta Llama 2 70B does not make a statistically significant performance difference, for all other group sizes and LLMs, adding the LLM to the human group increases performance. Posteriors are modeled as Student-t distributions based on 10-times repeated 5-fold cross-validation results, using (2) and applying the Nadeau-Bengio heuristic correction (3) to account for data overlap. After each label, the respective posterior is summarized using the posterior mean and a 95% equal-tailed credible interval.

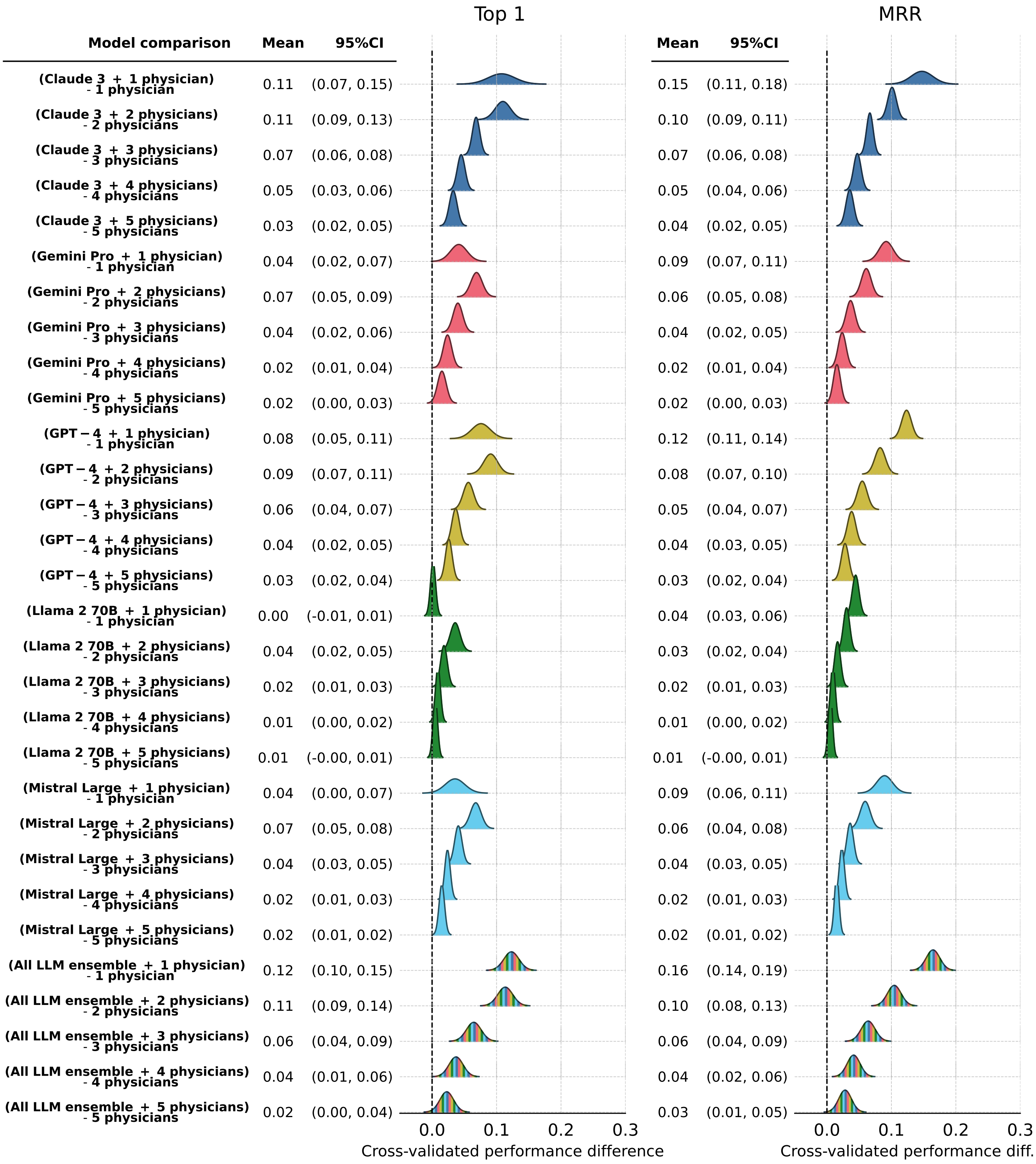

Fig. S12. Bayesian posteriors of cross-validated performance differences between hybrid ensembles and human-only ensembles. Hybrid ensembles, combining LLMs with one or more physicians, consistently outperform human-only ensembles in top-1 accuracy and MRR. Only combining a single physician with Meta Llama 2 70B yields no statistically superior performance for top-1, for all other group sizes and LLMs, adding the LLM to the human group increases performance. Posteriors are modeled as Student-t distributions based on 10-times repeated 5-fold cross-validation results, using (2) and applying the Nadeau-Bengio heuristic correction (3) to account for data overlap. After each label, the respective posterior is summarized using the posterior mean and a 95% equal-tailed credible interval.

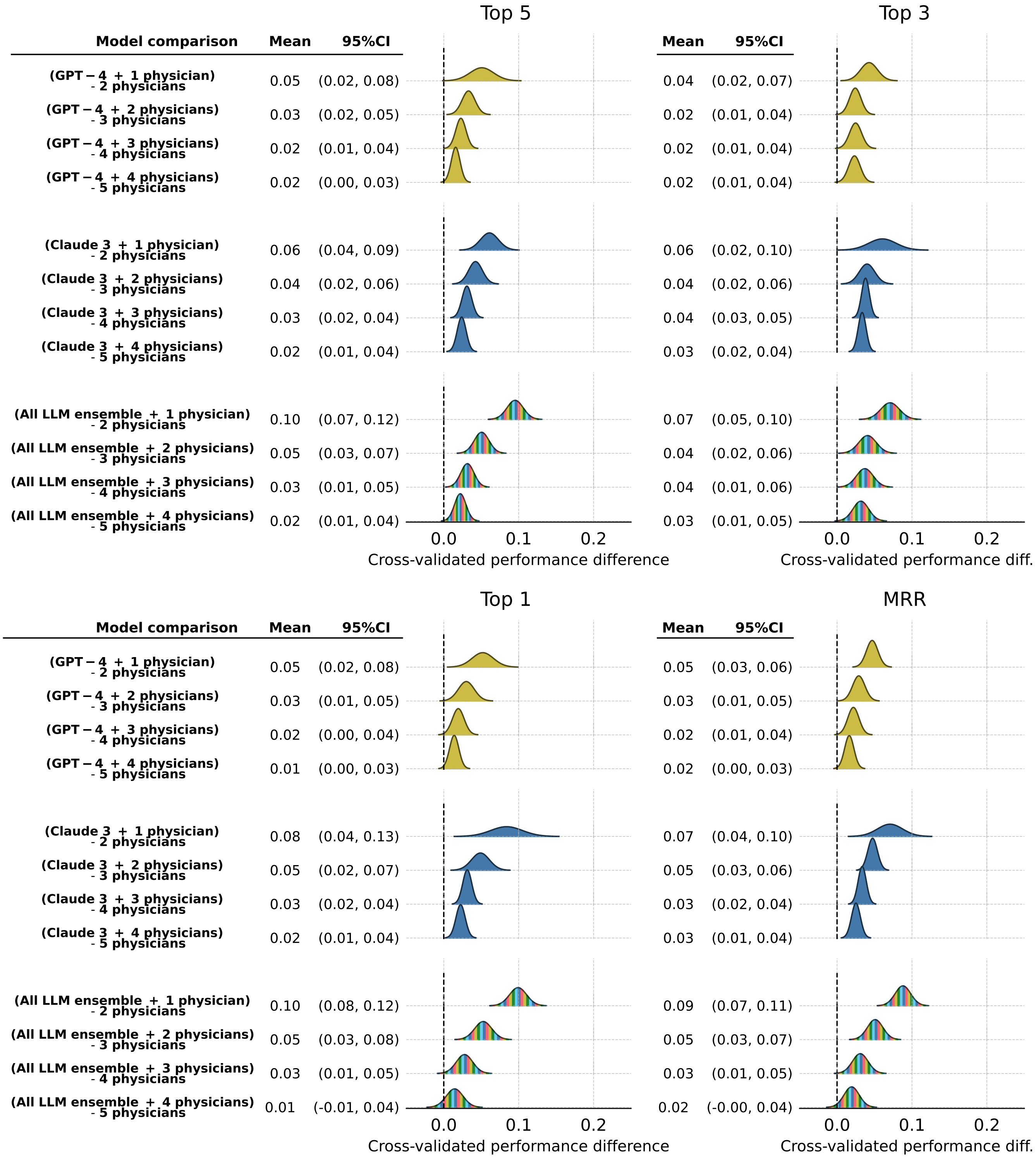

Fig. S13. Posteriors of cross-validated performance differences when adding either an LLM or a physician to a collective of physicians. Hybrid ensembles, which combine one or more physicians with LLMs, consistently outperform human-only ensembles where an additional physician is added instead of an LLM. Posteriors are modeled as Student-t distributions based on 10-times repeated 5-fold cross-validation results, using (2) and applying the Nadeau-Bengio heuristic correction (3) to account for data overlap.

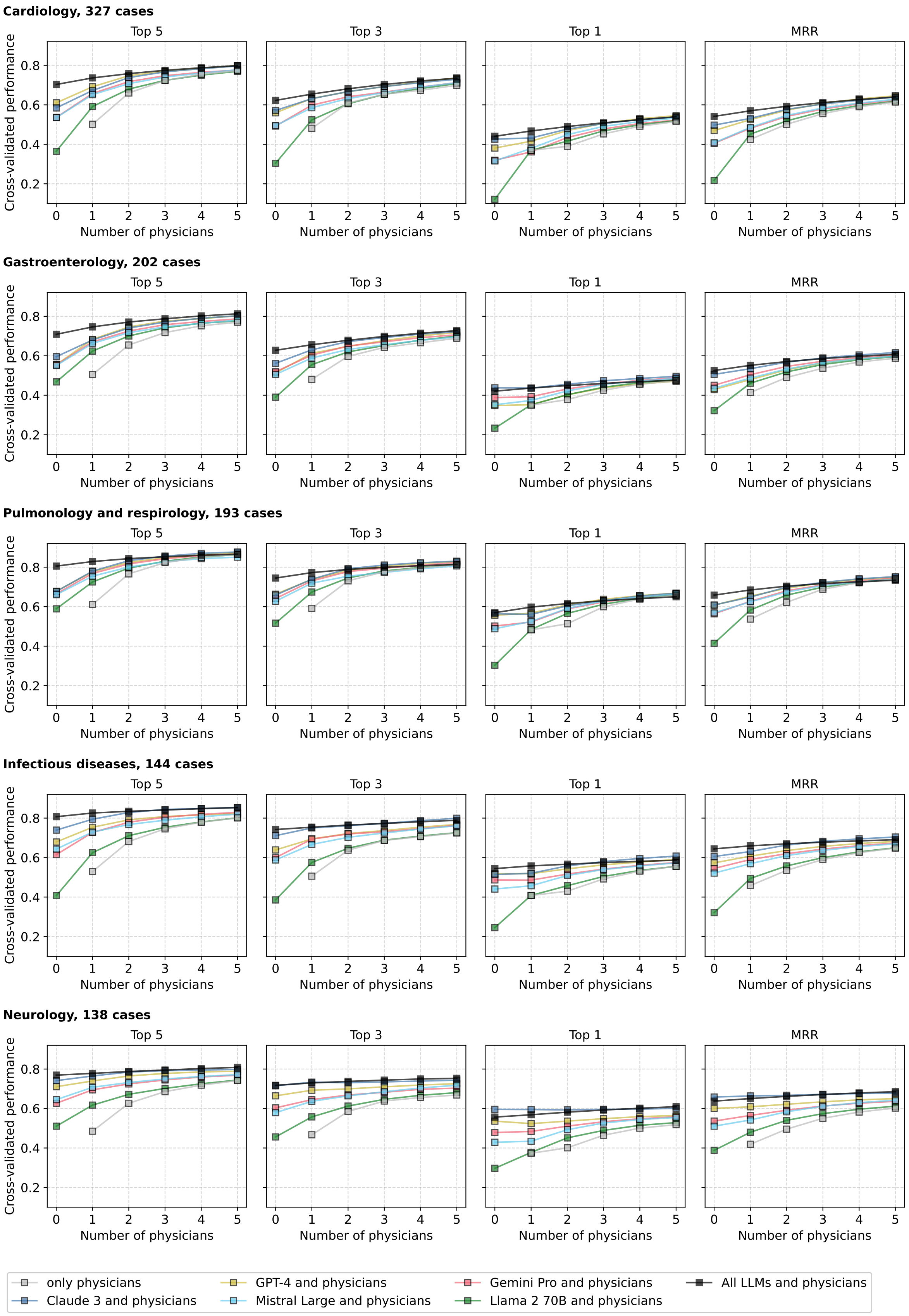

Fig. S14. Cross-validated performance of human-only ensembles and hybrid ensembles of humans and LLMs for the five most common specialties in the dataset. Across all medical specialties, combining humans and LLMs increased diagnostic accuracy relative to individual humans or LLMs. Increasing the number of humans in the ensemble generally increased performance. The best top-5 and top-3 accuracies were achieved by adding all LLMs to the ensemble, the best top-1 or MRR performance was achieved by adding either all LLMs or, in some cases, the best-performing individual LLM.

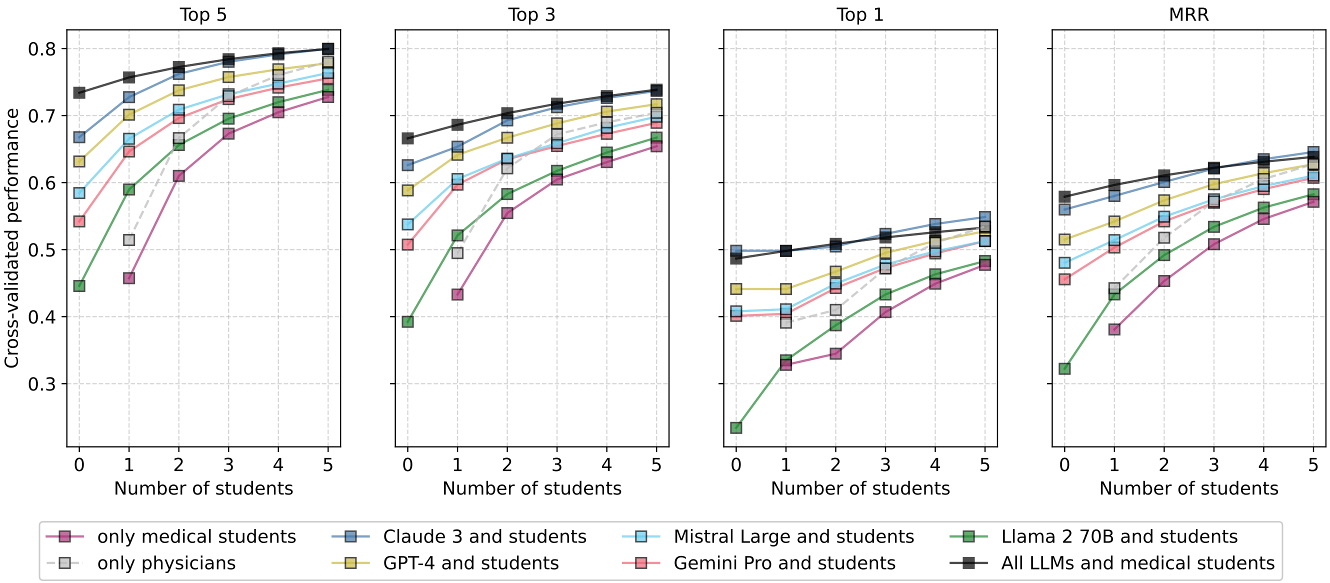

Fig. S15. Cross-validated performance of medical-student-only ensembles and hybrid ensembles of medical students and LLMs. Panels show performance for four outcome metrics (y axes): Top-k indicates the proportion of cases for which the correct diagnosis was among the k top-ranked diagnoses (for $k = \{1, 3, 5\}$); MRR shows the mean reciprocal rank of correct diagnoses across cases (see main text eq. 1). The individual performance of the five LLMs (and their combined performance in an all-LLMs ensemble) is shown as the left-most square of each color in each panel. The x axis shows the number of medical students added to individual LLMs or an all-LLMs ensemble. For comparison, the gray boxes and lines show the performance of physicians on the same set of cases. Results are based on 974 medical cases, each diagnosed by at least five medical students.

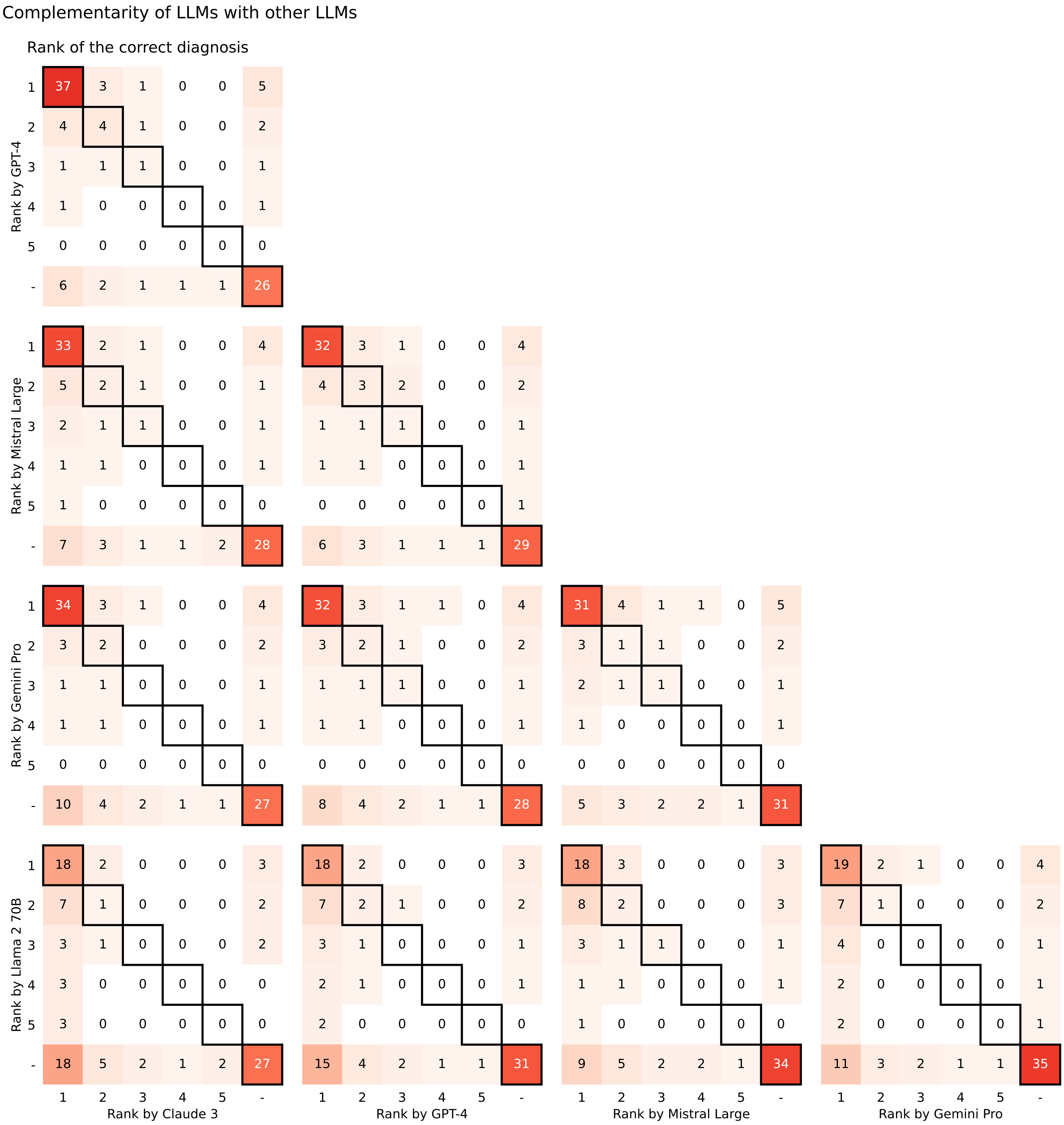

Fig. S16. Complementarity of solutions among LLMs: Rank of correct diagnoses within the differential diagnoses of LLMs. Panels show, for each of the ten possible pairs of LLMs, matrices with the percentages of cases for all 36 combinations of the LLM at the top (x axis) and LLMs on the side (y axis) assigning the correct diagnosis a particular rank (i.e., rank 1, 2, 3, 4, 5, or not ranked). The highlighted diagonal shows cases where LLMs assigned the same rank to the correct diagnosis. Results were extracted from the cross-validation procedure by recording the frequencies with which the LLMs assigned the correct diagnosis the same or a different rank, averaged across all cases and the five folds (see Methods). Note that due to rounding to integers, there may be small inconsistencies when summing rows or columns across matrices.

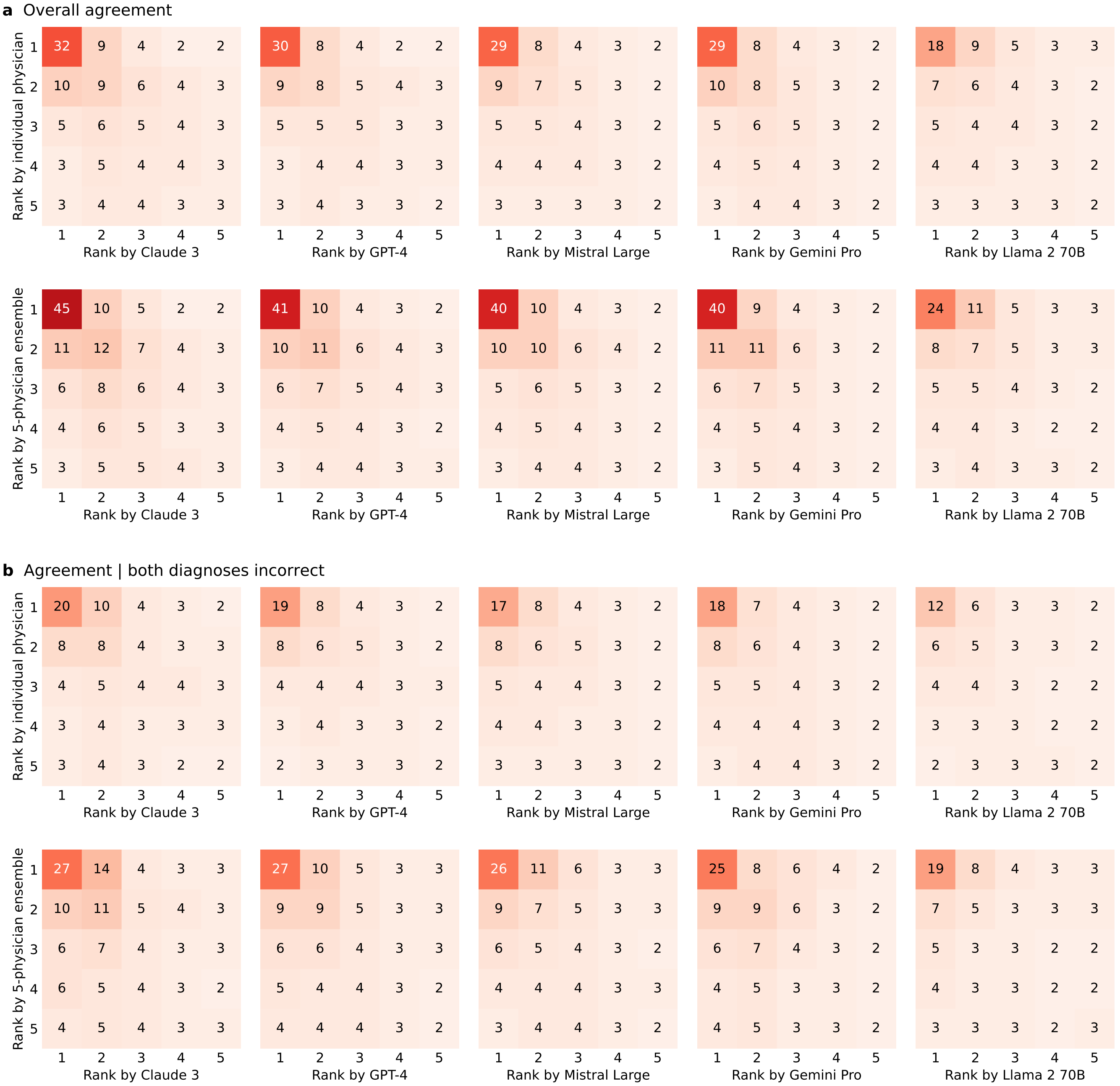

Fig. S17. Agreement among physicians and LLMs. Panels show the percentage of cases in which the same diagnoses were assigned to a particular rank combination, comparing individual physicians and 5-physician ensembles to LLMs. **a**, Overall agreement, regardless of whether the correct diagnosis was included in a particular rank combination. **b**, Agreement when both diagnosticians were incorrect for a rank combination. Results were extracted from the cross-validation procedure by recording the frequencies with which physicians and LLMs assigned the same diagnosis to a rank combination, averaged across all cases and the five folds (see Methods).

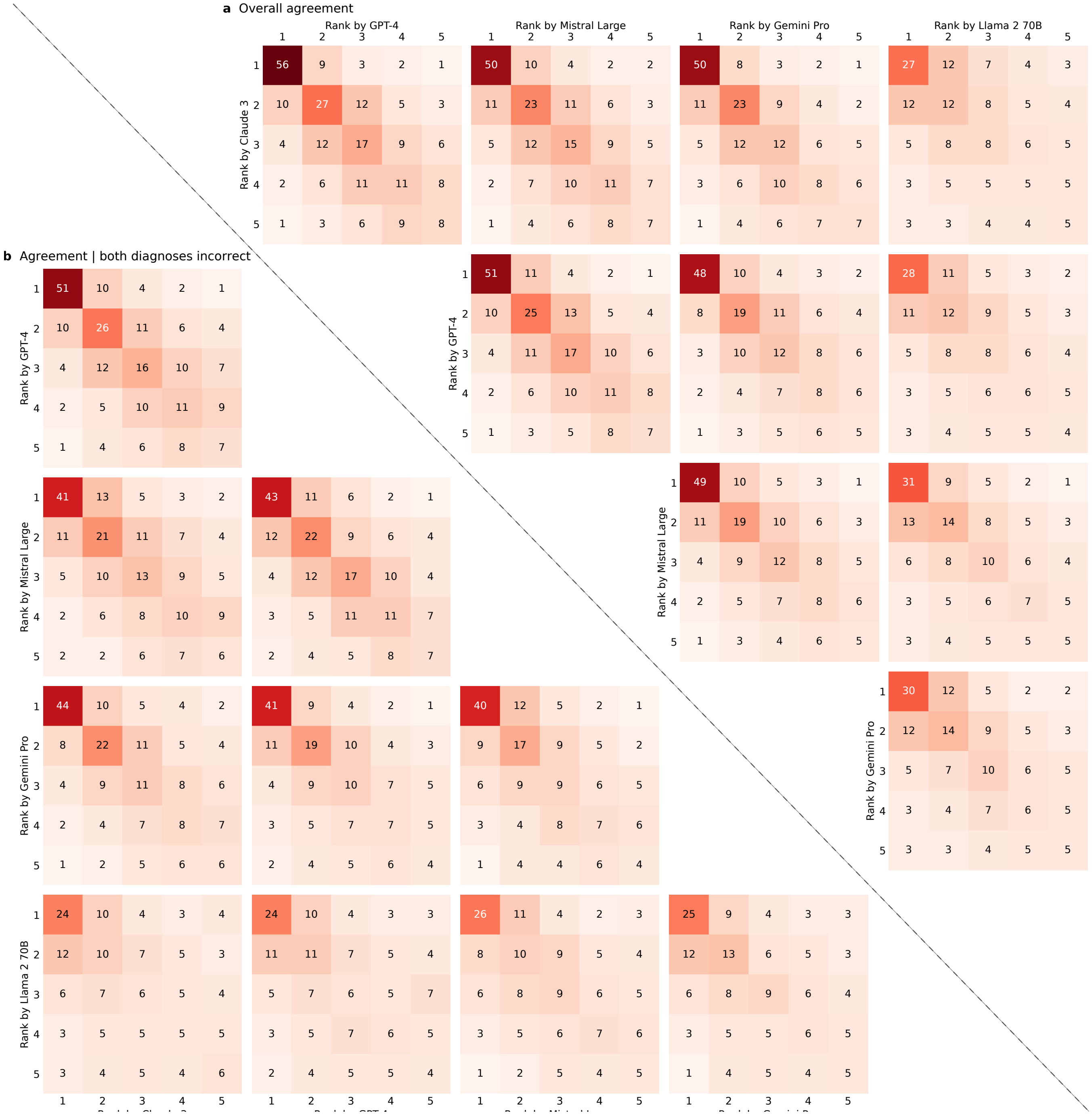

Fig. S18. Agreement among LLMs Panels show the percentage of cases in which the same diagnoses were assigned to a particular rank combination, comparing different LLMs to each other. **a**, Overall agreement, regardless of whether the correct diagnosis was included in a particular rank combination. **b**, Agreement when both LLMs were incorrect for a rank combination. Results were extracted from the cross-validation procedure by recording the frequencies with LLMs assigned the same diagnosis to a rank combination, averaged across all cases and the five folds (see Methods).

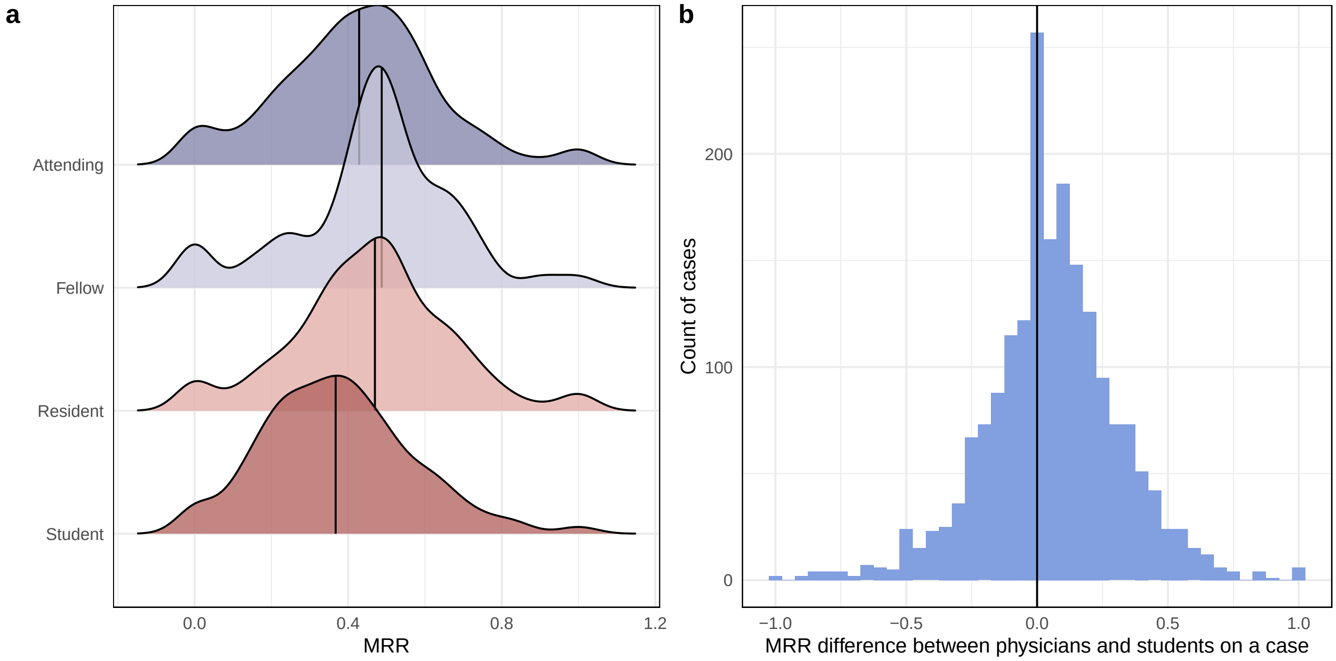

Fig. S19. Comparison of individual human performance across tenure levels. a, Density estimates of individual MRR values (x axis) for humans who solved at least five cases by tenure level. Vertical lines represent median MRR values within the tenure level. As the performance distributions of the three most experienced tenure levels (i.e., attending physicians, fellows, and resident physicians, representing senior doctors, doctors undergoing specialized training, and doctors in training, respectively) were similar, we combined these three groups. **b**, Comparing the pooled performance of physicians with that of medical students. For each case, the mean MRR across all physicians and all medical students was calculated. The distribution shows the difference in MRR between the two groups at the case level, with positive (negative) values indicating higher MRR for physicians (students). Physicians and students showed no difference in MRR in many cases, but the MRR for physicians was generally higher MRR than that for medical students.

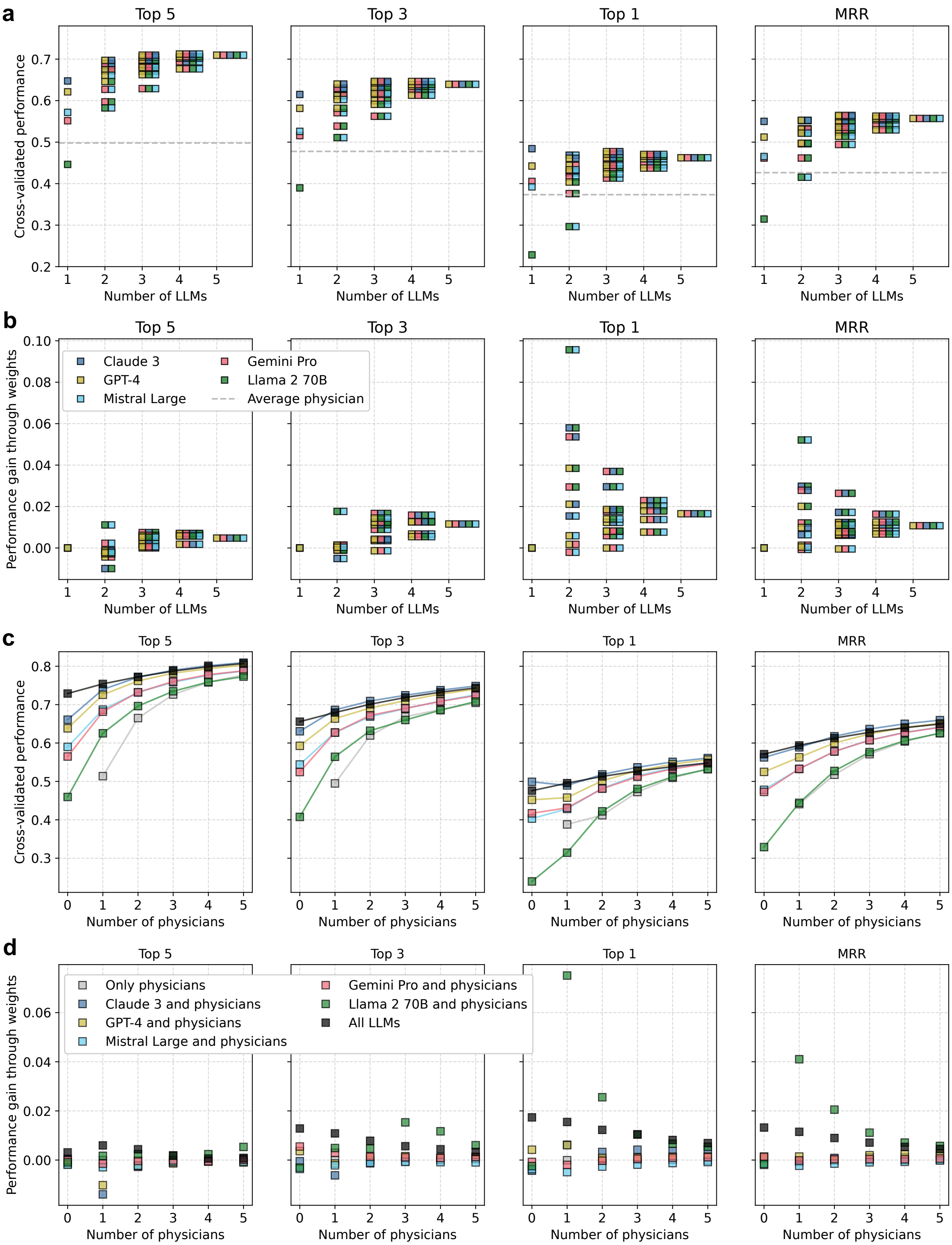

Fig. S20. Effect of weighting on cross-validated performance of LLM and hybrid human–LLM ensembles. a, Cross-validated performance (y axis) of five individual LLMs and ensembles of all possible combinations of LLMs with equal weights applied in the aggregation step. **b**, Signed difference in performance between weighted and unweighted aggregation (y axis); higher values indicate greater gains from applying weights. **c**, Cross-validated performance of hybrid physician-LLM ensembles with equal weights applied in the aggregation step (y axis). **d**, Signed difference in performance between weighted and unweighted aggregation for hybrid human–LLM ensembles. For most LLM- and hybrid ensembles, using the Weighted Majority Voting Ensemble approach (1) increased diagnostic accuracy, particularly for top-1 and MRR.

Table S1. LLM performance per prompt and accuracy metric on the whole dataset of 2,133 cases. Note that in the cross-validation results presented in the main text, prompts were selected on a subset (one fold) of these cases.

Prompt	MRR	Top 1	Top 3	Top 5
**Claude 3**				
base__common	0.499	0.441	0.557	0.585
base__common__fewshot	0.558	0.494	0.622	0.650
base__common__selfconsistent	0.509	0.449	0.568	0.594
base__common__selfconsistent__fewshot	0.554	0.490	0.619	0.645
base__impersonation__common	0.492	0.433	0.550	0.578
base__impersonation__common__fewshot	0.553	0.488	0.617	0.646
base__impersonation__common__selfconsistent	0.501	0.440	0.562	0.586
base__impersonation__common__selfconsistent__fewshot	0.557	0.492	0.622	0.651
base__impersonation__sct	0.454	0.379	0.526	0.568
base__impersonation__sct__fewshot	0.543	0.471	0.607	0.654
base__impersonation__sct__selfconsistent	0.469	0.396	0.542	0.579
base__impersonation__sct__selfconsistent__fewshot	0.539	0.465	0.608	0.649
base__sct	0.453	0.379	0.525	0.565
base__sct__fewshot	0.549	0.478	0.620	0.658
base__sct__selfconsistent	0.460	0.388	0.529	0.571
base__sct__selfconsistent__fewshot	0.542	0.468	0.613	0.651
**Gemini Pro**				
base__common	0.386	0.339	0.430	0.458
base__common__fewshot	0.462	0.411	0.508	0.539
base__common__selfconsistent	0.391	0.346	0.432	0.460
base__common__selfconsistent__fewshot	0.466	0.413	0.513	0.548
base__impersonation__common	0.391	0.343	0.436	0.463
base__impersonation__common__fewshot	0.450	0.395	0.499	0.533
base__impersonation__common__selfconsistent	0.398	0.350	0.443	0.470
base__impersonation__common__selfconsistent__fewshot	0.457	0.401	0.505	0.545
base__impersonation__sct	0.402	0.328	0.472	0.515
base__impersonation__sct__fewshot	0.465	0.407	0.519	0.557
base__impersonation__sct__selfconsistent	0.413	0.341	0.482	0.522
base__impersonation__sct__selfconsistent__fewshot	0.465	0.403	0.523	0.559
base__sct	0.406	0.335	0.472	0.512
base__sct__fewshot	0.470	0.412	0.523	0.559
base__sct__selfconsistent	0.423	0.354	0.487	0.529
base__sct__selfconsistent__fewshot	0.468	0.408	0.523	0.558
**Llama 2 70B**				
base__common	0.311	0.225	0.387	0.446
base__common__fewshot	0.271	0.228	0.305	0.339
base__common__selfconsistent	0.315	0.231	0.389	0.450
base__common__selfconsistent__fewshot	0.285	0.224	0.335	0.377
base__impersonation__common	0.296	0.211	0.373	0.431
base__impersonation__common__fewshot	0.278	0.217	0.331	0.372
base__impersonation__common__selfconsistent	0.303	0.215	0.383	0.440
base__impersonation__common__selfconsistent__fewshot	0.284	0.208	0.348	0.401
base__impersonation__sct	0.305	0.221	0.381	0.438
base__impersonation__sct__fewshot	0.231	0.146	0.310	0.364
base__impersonation__sct__selfconsistent	0.312	0.228	0.388	0.446
base__impersonation__sct__selfconsistent__fewshot	0.246	0.154	0.332	0.386
base__sct	0.312	0.228	0.386	0.446
base__sct__fewshot	0.228	0.164	0.287	0.331
base__sct__selfconsistent	0.323	0.238	0.401	0.455
base__sct__selfconsistent__fewshot	0.263	0.184	0.335	0.386
**Mistral Large**				
base__common	0.428	0.347	0.505	0.546
base__common__fewshot	0.468	0.395	0.532	0.581
base__common__selfconsistent	0.434	0.353	0.511	0.550

Continued on next page

Table S1 – continued from previous page

Prompt	MRR	top1	top3	top5
base_common_selfconsistent_fewshot	0.467	0.395	0.532	0.580
base_impersonation_common	0.428	0.356	0.493	0.536
base_impersonation_common_fewshot	0.460	0.390	0.525	0.570
base_impersonation_common_selfconsistent	0.428	0.358	0.493	0.532
base_impersonation_common_selfconsistent_fewshot	0.463	0.391	0.528	0.577
base_impersonation_sct	0.321	0.237	0.400	0.451
base_impersonation_sct_fewshot	0.445	0.373	0.505	0.561
base_impersonation_sct_selfconsistent	0.326	0.244	0.409	0.450
base_impersonation_sct_selfconsistent_fewshot	0.446	0.372	0.508	0.566
base_sct	0.347	0.256	0.435	0.484
base_sct_fewshot	0.447	0.372	0.512	0.568
base_sct_selfconsistent	0.355	0.261	0.442	0.498
base_sct_selfconsistent_fewshot	0.449	0.374	0.515	0.570
**GPT-4**				
base_common	0.478	0.412	0.541	0.577
base_common_fewshot	0.519	0.451	0.584	0.616
base_common_selfconsistent	0.487	0.423	0.548	0.580
base_common_selfconsistent_fewshot	0.516	0.446	0.584	0.618
base_impersonation_common	0.462	0.396	0.527	0.557
base_impersonation_common_fewshot	0.503	0.434	0.570	0.600
base_impersonation_common_selfconsistent	0.471	0.409	0.531	0.562
base_impersonation_common_selfconsistent_fewshot	0.509	0.443	0.575	0.606
base_impersonation_sct	0.410	0.329	0.486	0.531
base_impersonation_sct_fewshot	0.504	0.425	0.575	0.623
base_impersonation_sct_selfconsistent	0.433	0.356	0.502	0.544
base_impersonation_sct_selfconsistent_fewshot	0.512	0.433	0.584	0.628
base_sct	0.418	0.338	0.491	0.537
base_sct_fewshot	0.516	0.440	0.587	0.630
base_sct_selfconsistent	0.443	0.367	0.514	0.555
base_sct_selfconsistent_fewshot	0.517	0.438	0.592	0.631

References

1. A Dogan, D Birant, A weighted majority voting ensemble approach for classification in *2019 4th International Conference on Computer Science and Engineering (UBMK).* (IEEE, Samsun, Turkey), (2019).
2. A Benavoli, G Corani, J Demšar, M Zaffalon, Time for a Change: A Tutorial for Comparing Multiple Classifiers Through Bayesian Analysis. *J. Mach. Learn. Res.* **18**, 1–36 (2017).
3. C Nadeau, Y Bengio, Inference for the generalization error. *Mach. Learn.* **52**, 239–281 (2003).
